# Drug repurposing screening and mechanism analysis based on human colorectal cancer organoids

**DOI:** 10.1093/procel/pwad038

**Published:** 2023-06-22

**Authors:** Yunuo Mao, Wei Wang, Jingwei Yang, Xin Zhou, Yongqu Lu, Junpeng Gao, Xiao Wang, Lu Wen, Wei Fu, Fuchou Tang

**Affiliations:** School of Life Sciences, Biomedical Pioneering Innovation Center, Department of General Surgery, Third Hospital, Peking University, Beijing 100871, China; Beijing Advanced Innovation Center for Genomics (ICG), Ministry of Education Key Laboratory of Cell Proliferation and Differentiation, Beijing 100871, China; The Research Center of Stem Cell and Regenerative Medicine, School of Basic Medical Sciences, Cheeloo College of Medicine, Shandong University, Jinan 250012, China; School of Life Sciences, Biomedical Pioneering Innovation Center, Department of General Surgery, Third Hospital, Peking University, Beijing 100871, China; Beijing Advanced Innovation Center for Genomics (ICG), Ministry of Education Key Laboratory of Cell Proliferation and Differentiation, Beijing 100871, China; School of Life Sciences, Biomedical Pioneering Innovation Center, Department of General Surgery, Third Hospital, Peking University, Beijing 100871, China; Beijing Advanced Innovation Center for Genomics (ICG), Ministry of Education Key Laboratory of Cell Proliferation and Differentiation, Beijing 100871, China; School of Life Sciences, Biomedical Pioneering Innovation Center, Department of General Surgery, Third Hospital, Peking University, Beijing 100871, China; Peking University Third Hospital Cancer Center, Beijing 100871, China; School of Life Sciences, Biomedical Pioneering Innovation Center, Department of General Surgery, Third Hospital, Peking University, Beijing 100871, China; Peking University Third Hospital Cancer Center, Beijing 100871, China; School of Life Sciences, Biomedical Pioneering Innovation Center, Department of General Surgery, Third Hospital, Peking University, Beijing 100871, China; Beijing Advanced Innovation Center for Genomics (ICG), Ministry of Education Key Laboratory of Cell Proliferation and Differentiation, Beijing 100871, China; School of Life Sciences, Biomedical Pioneering Innovation Center, Department of General Surgery, Third Hospital, Peking University, Beijing 100871, China; School of Life Sciences, Biomedical Pioneering Innovation Center, Department of General Surgery, Third Hospital, Peking University, Beijing 100871, China; Beijing Advanced Innovation Center for Genomics (ICG), Ministry of Education Key Laboratory of Cell Proliferation and Differentiation, Beijing 100871, China; School of Life Sciences, Biomedical Pioneering Innovation Center, Department of General Surgery, Third Hospital, Peking University, Beijing 100871, China; Peking University Third Hospital Cancer Center, Beijing 100871, China; School of Life Sciences, Biomedical Pioneering Innovation Center, Department of General Surgery, Third Hospital, Peking University, Beijing 100871, China; Beijing Advanced Innovation Center for Genomics (ICG), Ministry of Education Key Laboratory of Cell Proliferation and Differentiation, Beijing 100871, China; Peking-Tsinghua Center for Life Sciences, Peking University, Beijing 100871, China

**Keywords:** colorectal cancer, organoids, drug repurposing, patient-derived organoids-based xenograft, mechanism of action

## Abstract

Colorectal cancer (CRC) is a highly heterogeneous cancer and exploring novel therapeutic options is a pressing issue that needs to be addressed. Here, we established human CRC tumor-derived organoids that well represent both morphological and molecular heterogeneities of original tumors. To efficiently identify repurposed drugs for CRC, we developed a robust organoid-based drug screening system. By combining the repurposed drug library and computation-based drug prediction, 335 drugs were tested and 34 drugs with anti-CRC effects were identified. More importantly, we conducted a detailed transcriptome analysis of drug responses and divided the drug response signatures into five representative patterns: differentiation induction, growth inhibition, metabolism inhibition, immune response promotion, and cell cycle inhibition. The anticancer activities of drug candidates were further validated in the established patient-derived organoids-based xenograft (PDOX) system *in vivo*. We found that fedratinib, trametinib, and bortezomib exhibited effective anticancer effects. Furthermore, the concordance and discordance of drug response signatures between organoids *in vitro* and pairwise PDOX *in vivo* were evaluated. Our study offers an innovative approach for drug discovery, and the representative transcriptome features of drug responses provide valuable resources for developing novel clinical treatments for CRC.

## Introduction

Colorectal cancer (CRC) is one of the most commonly diagnosed malignant cancers worldwide, with an increasing incidence and mortality (Sung et al., 2021). By 2030, the burden of CRC is predicted to increase by 60% (Arnold et al., 2017). As the research on CRC continue to progress, increasing targeted drugs and therapeutic regimens were successfully developed and proven to be effective in the clinical treatments of CRC. For example, patients carrying BRAF^V600E^ mutation can benefit significantly from the targeted therapy (Sveen et al., 2020). Also, KRAS/BRAF-wild metastatic colorectal cancer (mCRC) patients’ survival can be largely extended by the combination of anti-EGFR therapy and chemotherapy (Biller and Schrag, 2021). Since CRC is a highly heterogeneous and complex disease, patients with different molecular characteristics often respond very differently to the same treatment strategy. Especially, during the progression of cancer, tumor cells tend to acquire different signatures and generally become more heterogeneous. Therefore, although targeted therapies have significantly improved the overall survival of mCRC, there is still a large number of patients lacking effective targeted drugs or exhibiting drug resistance during treatment. Given the prevalence of CRC and limitation in their treatments, improving the current clinical approaches and developing new therapeutic agents are therefore imperative.

Drug repurposing for cancer therapy is a promising strategy for drug discovery. In comparison with *de novo* drug development, drug repurposing as an ideal replacement, significantly shortens the time, cuts the investments, and improves the success rates of preclinical drug discovery (Gonzalez-Fierro and Duenas-Gonzalez, 2021). Anticancer drug repurposing has moved from the “pre-genomic era” to the “genomic era”. Instead of discovering drugs empirically, integrating with the existing drug signature databases [CTRP (Basu et al., 2013; Seashore-Ludlow et al., 2015), CMap (Lamb et al., 2006; Subramanian et al., 2017), GDSC (Yang et al., 2013), etc.] and disease signature transcriptomic databases (such as TCGA) to predict the potential drugs enable the discovery process to be more efficient. Moreover, combining them with additional experimental approaches such as phenotypic screening would improve the identification of repurposed drugs (Pushpakom et al., 2019). However, the current drug screening is mainly based on traditional cancer cell lines, which experience genome changes such as genetic drift after long-term *in vitro* culture and lose the original molecular characteristics of the parental *in vivo* tumors eventually.

Patient-derived organoids provide an ideal preclinical model for cancer research, which faithfully recapitulates the molecular characteristics and the heterogeneities of parental tumor tissues (van de Wetering et al., 2015; Broutier et al., 2017; Sachs et al., 2018; Yan et al., 2018; Kopper et al., 2019). Recently, many studies have shown that organoids can precisely predict the patients’ responses to targeted therapies and chemotherapies (van de Wetering et al., 2015; Ooft et al., 2019; Yao et al., 2020; Yin et al., 2020). CRC organoids have been successfully established and the culture conditions have been well evaluated (van de Wetering et al., 2015; Fujii et al., 2016; Wang et al., 2022). Several studies have utilized the organoid-based platform to conduct anticancer drug screening. Nevertheless, the screening assay is mainly based on the measurement of cell growth, and the biological mechanisms of drugs underlying the anticancer effects remain largely elusive. Furthermore, the anticancer mechanisms of the drugs have been sparsely described at the *in vivo* level.

In order to efficiently discover repurposed drugs for CRC, we first established long-term stably cultured, highly representative CRC organoids. The consistencies between organoids and parental tumors have been well confirmed by multi-omics sequencing approaches. We then established a robust organoid-based screening platform, a total of 335 approved small-molecule drugs and computationally predicted drug candidates were tested. According to the drug inhibitory efficiencies, 34 drug candidates were successfully selected out and validated to be effective in killing CRC organoids. Notably, by taking advantage of RNA-seq, we described the biological mechanisms of drugs and classified the drug response patterns of CRC organoids into five prevalent and representative groups, which represent the general responding features of CRC to different drugs. Furthermore, the therapeutic capacities of the screened drug candidates were validated in patient-derived organoids-based xenograft (PDOX) models *in vivo*. Trametinib, bortezomib, and fedratinib displayed powerful tumor inhibitory effects, which is comparable to 5-fluorouracil (5fu), the first-line drug for clinical treatments of CRC. In addition, the similarity and discordance of drug response features were well evaluated between organoids *in vitro* and pairwise PDOX *in vivo*. We also evaluated the feasibility of applying our CRC organoid culture platform to explore potential drug combination therapy and found that the bortezomib and fedratinib have potential synergistic effects for killing CRC organoids.

In summary, we conducted robust organoid-based drug screening, profiled drug responding signatures of CRC organoids, identified potential repurposed drugs, and assessed the *in vitro* and *in vivo* drug screening system based on transcriptome features, which provided a sequencing-based drug identification strategy that will accelerate the development of clinical trials of CRC.

## Results

### Long-term cultured human CRC organoids remain the histological features and molecular characteristics of the parental tumors

**Figure 1.**
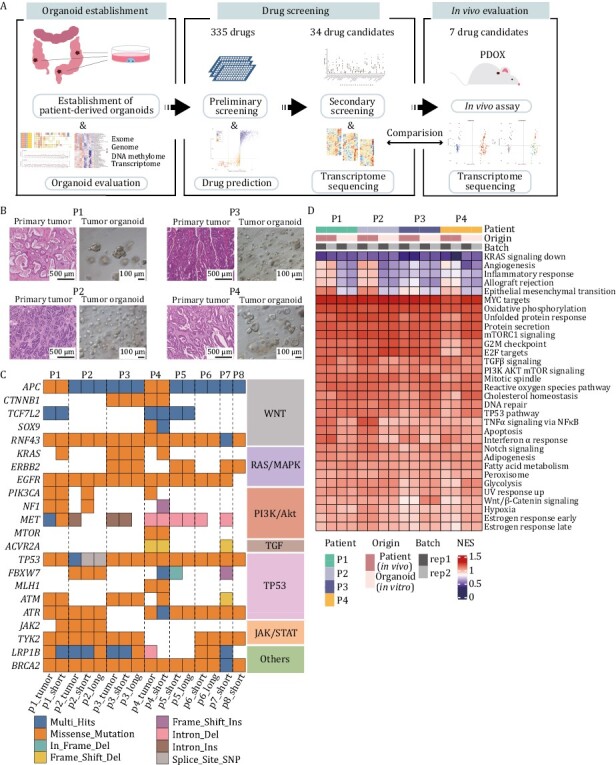
**Colorectal tumor organoids preserve the histological architecture and genomic characterization of primary tissues.** (A) Experimental workflow. Mainly include establishing CRC organoids, evaluating the culture system, screening repositioned drugs, analyzing drug mechanisms and validating drug efficiency *in vivo*. (B) H&E staining of four patients’ primary tumors (left) and brightfield of corresponding organoids (right). (C) Comparison of the gene mutations of eight original tumor tissues with corresponding organoids cultured in short-term (1 week) or long-term (~2 months). Different color represents different types of mutations. (D) Expression levels of cancer-related pathways of four patient tumor tissues and corresponding organoids.

To identify repurposed therapeutic agents for CRC, we designed experiments that combined organoid-based drug screening and transcriptome sequencing-based evaluation (Fig. 1A). First, we established a human CRC organoid culture system based on the previously published protocols (see Methods). With several modifications of the culture medium, a total of eight tumor organoid lines were successfully derived from eight patients with different types of CRCs (Table S1). All of the organoids could stably maintain long-term expansion capacities (>3 months), with a 1:4 or 1:5 passage ratio. We observed that the morphologies of patient tumor tissues differed from each other, and corresponding organoids represented the heterogeneous morphologies of tumor tissues (Figs. 1B and S1A).

To further characterize the molecular features, we assessed the concordance between organoids and parental tumor tissues at the multi-omics level. As for the single nucleotide variations (SNVs), the whole-exome sequencing (WES) provided a global profile of tumor-specific genetic mutations. We identified key driver mutations in both organoids and corresponding tumor tissues, which are frequently mutated genes in CRC. These mutations are classified into different types according to the signaling pathways or related genes, such as Wnt/β-catenin, RAS/MAPK, PI3K/AKT, TGF, TP53-related pathways, JAK/STAT. Key driver genes of CRC such as *APC* and *TP53* are two common mutations detected in CRC, which were identified in all these eight organoid lines we established. Three out of eight organoid lines harbored the well-known mutations of *KRAS*. In addition to these driver mutated genes, other mutated genes of different signaling pathways were also retained in organoids compared to pairwise tumor tissues *in vivo*. We also captured one organoid derived from a microsatellite unstable patient (P4), which harbored *MLH1* and *ACVR2A* mutations (Fig. 1C). With respect to the maintenance of the key gene mutations, these cultured organoids can well recapitulate the genetic signatures of tumors *in vivo*. To investigate the conservation of mutations during long-term culture, four organoid lines were additionally analyzed by WES after 2 months of culture, and it was shown that mutations were in general sustained, which can be presented by mutations of *TP53*, *EGFR*, *BRCA2*, etc. in P2, P3 and P6 (Figs. 1C and S1B). Also, the high Jaccard index of short- and long-term cultured organoids also confirmed the maintenance of mutations. Furthermore, by using whole genome sequencing (WGS), we found that the copy number variations (CNVs) of organoids cultured *in vitro* were well consistent with the corresponding tumor tissues *in vivo* (Fig. S1C). The above results analyzed a diverse range of genomic features of organoids which represent the heterogeneities of CRC patients and verified that organoids could faithfully maintain the mutations and CNV features of the corresponding tumor tissues of CRC patients.

At the transcriptome level, organoids well preserved the expression levels of tumor-related important signaling pathways, such as PI3K/AKT, Wnt/β-catenin and TP53 pathway (Fig. 1D). Furthermore, we integrated DNA methylome sequencing data and focused on the features of specific genes. It was found that the organoid cultured *in vitro* could largely maintain the DNA methylation patterns of gene promoter regions as those of *in vivo* tumor tissues, including the genes *BCL2L1*, *IMPDH2*, and *TGFBI*, which have been reported to be associated with CRC progression (Sillars-Hardebol et al., 2012; Duan et al., 2018; Chiavarina et al., 2021) (Fig. S1D). Collectively, these results demonstrate that the organoids we cultured well resembled the corresponding matching tumor tissues *in vivo*, including both the histological, genomic and epigenomic features, which is critical for the subsequent anticancer drug screening and interpretation of the drugs’ mechanisms of action (MOA).

### The prediction and screening of repurposed drugs to suppress CRC patient-derived organoids

**Figure 2.**
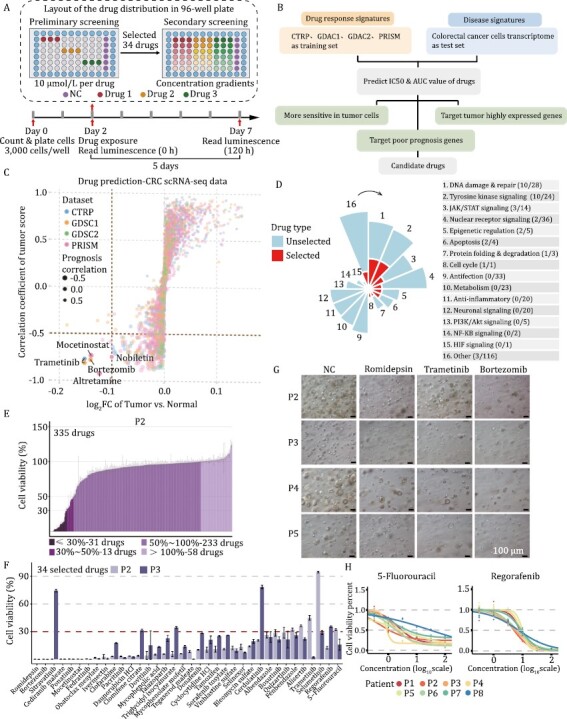
**Drug prediction and organoid-based drug screening.** (A) Schematic diagram displaying the experimental procedure of drug screening. Organoids were dissociated into single cells and were further seeded in 96-well culture plate (3,000 cells/well) at day 0. Drugs were added at day 2. 10 μmol/L of drugs were used in preliminary screening. 34 Drugs with great anti-tumor effects were selected out for secondary screening, and 2 positive control drugs (5-fluorouracil and regorafenib) were also used in secondary screening. The concentration gradients were set for the secondary screening. To get better interpretable value, drugs were diluted at 3.16 (half-log) times, 2 times, 5 times or 10 times respectively, depending on the sensitivity of the organoids’ response to the drugs. ATP values at the beginning and the end of drug treatment were measured respectively, which were used for the calculation of GR_50_. Different color stands for different drugs. Three replicates were set up for each drug treatment. (B) Drug prediction procedure. Three screening conditions were marked in green boxes. (C) Prediction results. Candidate drugs were marked in the figure. Different dot color represents drugs predicted from different databases. (D) Distribution of the total and selected drug-targeted pathways. The classification of 34 drug candidates was presented in red. (E) Preliminary screening result for patient 2-derived organoid. (F) Cell viability of selected 34 drugs and 2 positive control drugs (5-fluorouracil and regorafenib) in both patient 2- and 3-derived organoids. Different colors stand for different patient-derived organoids. (G) Representative brightfield of organoids exposed to vehicle (NC), romidepsin, trametinib, and bortezomib. Scale bars, 100 μm. (H) Drug dose-response curves of eight CRC patient-derived organoids treated with the two positive control drugs (5-fluorouracil and regorafenib). Each point indicates the mean value of three replicates.

Organoids we established displayed a closer recapitulation of tumor tissues and could serve as an ideal model for anticancer drug screening. In an attempt to identify potential drugs for CRC therapy effectively, we developed an approach that combined computation-based drug prediction with experiment-based drug screening using organoids. First of all, we established an organoid-based drug screening platform. To improve the reproducibility between different wells, we optimized the present drug screening protocols (Driehuis et al., 2020): organoids were dissociated into single-cell suspension and then seeded into 96-well plates after precise counting of cell numbers. After about 2 days, when the organoid cells re-formed into spherical structures, the drugs were added and the treatment was maintained for 5 days. Celltiter-Glo 3D was used to measure the ATP values of cells. Traditional criteria used to access drug responses such as IC_50_ have limitation that they vary widely with the changes of proliferation rates. Growth rate inhibition (GR) metrics has been reported to be a more desirable measure for *in vitro* drug screening, eliminating the disturbance of cell proliferation rate on drug sensitivity by normalizing growth rate inhibition of cells (Hafner et al., 2016). GR_50_ is the concentration of drugs at which GR value equals 0.5. Therefore, the GR_50_ value was calculated to represent the sensitivity of the drugs in our screening platform (Fig. 2A).

Then, we developed a computation-based drug prediction approach (see Methods). Recent progress in high throughput sequencing has provided a rich resource for disease research and drug discovery. Taking advantage of the available transcriptome database, we conducted a ridge regression-based approach to predict drugs with potential inhibitory effects on CRC (Fig. 2B). The drug response signatures obtained from CTRP, PRISM, GDSC1, and GDSC2 databases and the disease signatures obtained from TCGA were used as training sets and test sets respectively. Transcriptome signatures of malignant epithelial cells and normal epithelial cells from a published single-cell transcriptome dataset were used to predict the potential drug responses for CRC (Lee et al., 2020). Based on differences between malignant epithelial cells and normal epithelial cells as well as correlations of predicted IC_50_ values with tumor signatures and prognosis, five drugs including mocetinostat, trametinib, nobiletin, bortezomib, and altretamine were stringently screened out (Fig. 2C).

With the aim of discovering drugs with less adverse effects, we employed a compound library of clinically tested drugs that were barely used in CRC clinical trials according to the clinicaltrial.gov database (Table S2). A total of 335 drugs, including five drugs that were computationally predicted, were used for further organoid-based screening (Figs. 2D, S2A and S2B). All of these drugs have passed phase I clinical trial for at least one type of human disease. Due to the heterogeneities of organoids, we used organoid lines established from two representative patient cases of CRC (P2 and P3) for preliminary screening simultaneously. Drugs on either of these two different organoid lines with cell viability of less than 30% (5fu was used as positive control) after treatment were considered to have significant inhibitory effects on CRC organoid cells. Among these 335 drugs, 34 drugs were successfully screened out (Figs. 2E, 2F and S2C). As expected, 27 out of these 34 drugs were anticancer drugs, targeting DNA damage and repair, and tyrosine kinase signaling, etc. The remaining drugs, although initially developed for the treatment of non-cancer diseases, also showed significant suppression for CRC-derived organoids in our screening (Fig. S2B).

To further confirm the anticancer efficiency of the drugs for a diverse set of CRCs with different molecular types, we tested the drugs in all eight established organoid lines. We found that different patient-derived organoids showed diverse sensitivities to these 34 drug candidates (Fig. S2D and S2E). Triglycidyl isocyanurate, quizartinib, and fenbendazole were the three drugs that had the greatest response differences among different organoid lines, indicating the diverse responding signatures of different tumors to the same drug. Romidepsin, trametinib, and bortezomib were the drugs that had the greatest inhibition rates (Fig. 2G). 5-Fluorouracil and regorafenib, two drugs commonly used in CRC clinical treatment, were used as the positive controls in our study. Varying sensitivities of these two clinical agents were also observed. P4 organoid was the most sensitive to 5-fluorouracil, while P8 was the least sensitive (Fig. 2H).

### Patient-derived organoids showed strong heterogeneities of drug responses

**Figure 3.**
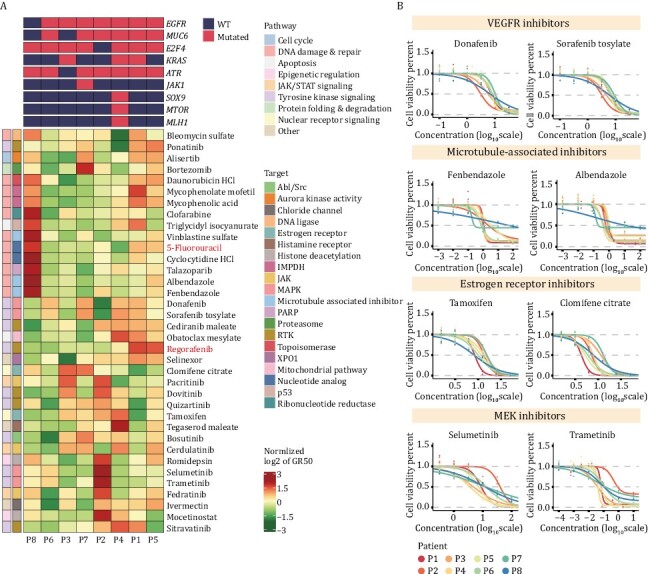
**Distinct heterogeneity of drug sensitivity was observed among different patient-derived organoids.** (A) Heatmap of normalized GR_50_ values for 34 drug candidates and 2 positive control drugs (5-fluorouracil and regorafenib) used to treat 8 CRC organoids. Green to red indicates sensitivity to insensitivity. (B) Dose-response curves displaying that the selected drugs with the same targets showed high consistency in drug sensitivity. Each point indicates the mean value of three replicates.

The organoids that we established well recapitulated the molecular characteristics of parental tumors and exhibited diverse gene mutations enabling us to observe the heterogeneities among different organoid lines. First, based on the drug sensitivity represented by the GR_50_ value, we observed that the sensitivities of different drugs with the same target were highly consistent across organoid lines, indicating the reliability of the drug screening system in our study (Fig. 3A and 3B). For instance, donafenib and sorafenib tosylate both target vascular endothelial growth factor receptor (VEGFR), and the sensitivity to both drugs showed high consistency among different organoid lines, with higher sensitivity in P2, P3 and P8 patient-derived organoid lines compared to other organoid lines. In addition, two anthelmintic drugs, fenbendazole, and albendazole, also showed similar drug sensitivities in eight lines of the organoids, with P8 being insensitive to both drugs. The same accordance was also observed in two estrogen receptor inhibitors (tamoxifen and clomifene citrate) and two MEK inhibitors (selumetinib and trametinib), confirming that our screening system provides a nice demonstration of inter-organoid (inter-patient) drug sensitivity. And these results also suggest that it is the inherent heterogeneities of biological features among different patient-derived organoid lines that lead to the differences in drug response profiles.

Next, we compared the differential drug sensitivities among patient-derived organoids. We found distinct differences in drug sensitivities for the same organoid line treated with different drugs, as well as different sensitivities among diverse organoid lines treated with the same drug, indicating the biological heterogeneities among different CRC patients (Figs. 3A and S3). Interestingly, we found that some patients were specifically insensitive to a class of drugs. For example, the P8 organoid line was very insensitive to drugs that target DNA damage and repair-related pathways. Moreover, the P2 organoid line displayed insensitive to MEK-target inhibitors, trametinib, and selumetinib.

### Drug response signatures were classified into five representative patterns based on transcriptomic characteristics

In an attempt to decipher the MOA of drug candidates that potentially inhibit CRC, we performed RNA-seq for organoids treated with all these 34 drug candidates we screened out as well as the two positive controls (5-fluorouracil and regorafenib). PCA analysis and correlation analysis revealed that organoids of different patient origins clustered individually, indicating that the differences in transcriptomes between organoids from different patients were greater than the differences induced by drugs (Fig. S4A and S4B). Moreover, we observed heterogeneities in drug responses for the same drug among different patient-derived organoid lines compared to organoids from the single patient (Fig. S4C). Therefore, to comprehensively reveal the MOA of these 34 drug candidates and exclude interference of the heterogeneities of drug responses, we integrated all the transcriptome data to explore the drug-induced changes in terms of the disturbance of tumor-related signaling pathways, expression of CRC characteristic genes, expression of drug resistance-related genes and the degree of cell differentiation.

**Figure 4.**
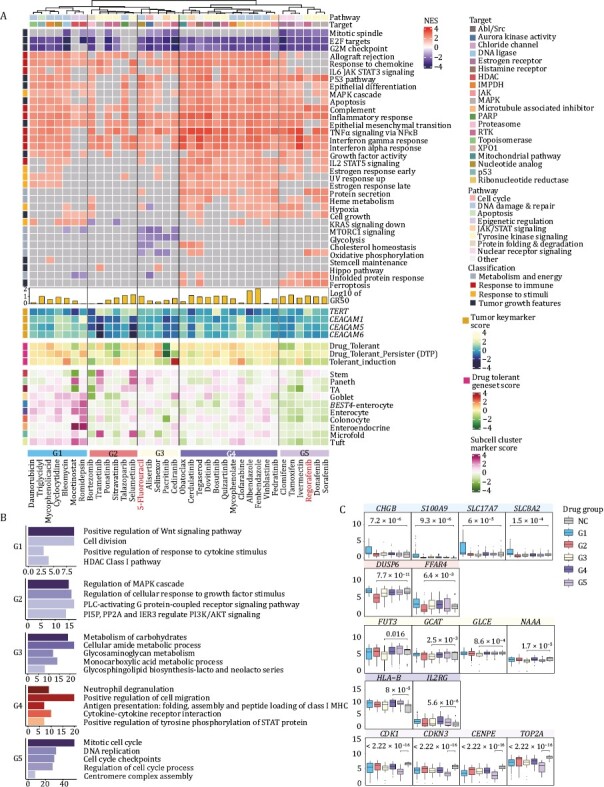
**Transcriptome characteristics of five distinct patterns of drug responses.** (A) Heatmap displaying the expression levels of drug-disturbed tumor-associated signaling pathways, characteristic genes of CRC, drug resistance-related genes and intestinal cell type marker genes of organoids exposed to 36 drugs [34 candidate drugs and 2 positive control drugs (5-fluorouracil and regorafenib)]. (B) Significantly enriched GO terms of five distinct drug response patterns. (C) Expression of genes characterizing five drug-response patterns. Boxplots detail the *P* values obtained (two-sided *t*-test). The centerline represents the median value, and the box range represents 1.5* the interquartile range.

Gene set enrichment analysis (GSEA) was performed to investigate the expression patterns of tumor-related signaling pathways in organoids treated with different drug candidates. We classified the tumor-related pathways into four groups based on their functions: cell proliferation, immune activation, stress response and metabolism. We found that almost all drugs (31 of 36) significantly inhibited cell cycle-related pathways, such as E2F targets and G2M checkpoints pathways, and 26 drugs exhibited the activation of apoptotic pathways. In addition, the disturbance of signaling pathways was consistent for drugs with the same targets, such as estrogen receptor inhibitors (clomifene and tamoxifen), histone deacetylation inhibitors (mocetinostat and romidepsin) and inhibitors related to inhibition of microtubule action (albendazole and fenbendazole), confirming that the accuracy of our transcriptome-based analysis for MOA signatures (Fig. 4A).

Interestingly, although all these 34 drug candidates demonstrated a significant suppressive effect against tumor organoids, different drugs interfered with different pathways, indicating that they acted in different ways to inhibit the viability of CRC organoids. According to the expression of the tumor-related signaling, we identified five distinct patterns of drug response signatures.

Pattern 1 (G1) was annotated as a differentiation induction group, which is characterized by higher expression of marker genes for different intestinal epithelial cell types. Gene function enrichment analysis revealed that downregulated genes mainly enriched for positive regulation of the WNT signaling pathway and cell division (Fig. 4B). And upregulated genes enriched for regulation of cell growth and cell adhesion (Fig. S5B). Meanwhile, *CHGB*, *S100A9*, *SLC17A7* and *SLC8A2*, which participated in intestinal absorption and secretion functions were upregulated significantly (Fig. 4C). *SLC2A9* and *SDHC*, low expression of which correlated with the worse survival of CRC, were significantly upregulated in this study (Fig. S5C). Moreover, it has been reported that the stemness of tumor cells correlates with tumor progression and metastasis, targeted which has great potential to induce tumor regression (Fumagalli et al., 2020). Thus, we speculated that drugs in G1 cluster may both inhibit the proliferation of tumor cells and promote cell differentiation. In addition, G1 contains two inhibitors targeting histone deacetylase (HDAC): mocetinostat and romidepsin. Both of the drugs have a stronger differentiation induction capability in tumor cells compared to other drugs in this class and have a greater ability to kill tumor cells (average GR_50_ values of these two drugs were 1.3 μmol/L and 3.7 nmol/L respectively). HDAC is known to repress chromatin opening and transcription factor binding by removing charged acetyl groups, which eventually affect cell growth. It has been reported that HDAC inhibitors promote the expression of genes involved in the synthesis of acetyl-coenzyme A (acetyl-CoA) from citrate and acetate (Srivatsan et al., 2020). In the present study, HDAC inhibitors’ treatment resulted in downregulation of the expression of *ACSS2* and *ACLY*, in agreement with the reported results, indicating that HDAC inhibitors interfered with acetyl-CoA synthesis and disturbed the function of HDAC (Fig. S5A). The above results showed that HDAC inhibitors were highly effective in inhibiting the viability of tumor cells, and consistent with the published work, HDAC inhibitors could stimulate the differentiation of intestinal epithelial cell types (Wang et al., 2017), which verified the reliability of the drug-response signatures based on the transcriptome analysis in this study.

Compounds in Pattern 2 (G2) significantly repressed the growth factor responses and the regulation of MAPK signaling pathways (Fig. 4B), so we identified G2 as a growth inhibition group. *DUSP6* and *FFAR4*, which are involved in the MAPK pathway, were downregulated (Fig. 4C). *DRD4* and *SLC9A3R2*, two genes associated with poor prognosis of CRC were downregulated (Fig. S5C).

Pattern 3 (G3) was identified as the metabolism inhibition group. Drugs in this cluster inhibited the expression of metabolism-related pathways of CRC organoids, particularly the glycolytic pathway. Genes downregulated in this cluster were enriched for functions such as carbohydrate metabolism and amino acid metabolism (Fig. 4B). Moreover, metabolism-related genes such as *FUT3*, *GCAT*, *GLCE*, and *NAAA* were clearly downregulated after drug treatment (Fig. 4C). Also, genes (*GRIA3* and *ENO2*) correlated with poor prognosis were observed downregulated in G3 (Fig. S5C). The positive control, 5-fluorouracil, a commonly used clinical drug for CRC treatment, was also in this group.

Pattern 4 (G4) exhibited an interesting signature that the immune response- and stress response-related pathways were activated. GSEA results revealed that the differentially expressed genes in this cluster were enriched in neutrophil degranulation, antigen presentation (Fig. 4A). This suggested that drugs in this class not only inhibit tumor cell proliferation but also promote the immune system to attack the tumor cells by enhancing the antigen presentation signatures of the tumor cells. Therefore, G4 was annotated as an immune response promoting group. *HLA-B* and *IL2RG*, genes participated in immune responses were significantly upregulated in G4 (Fig. 4C). Meanwhile, poor prognosis gene *ZEB1* was downregulated (Fig. S5C).

Pattern 5 (G5) was identified as the cell cycle inhibition group, which revealed the strongest phenotype displaying a decline in the cell cycle and DNA replication (Fig. 4B). In addition, genes that were upregulated in this group enriched for unfolded protein response pathways (Fig. S5B). A variety of markers of the cell cycle, such as *CDK1*, *CDKN3*, *CENPE*, and *TOP2A* were significantly downregulated (Fig. 4C). *PARD6G*, which was correlated with worse survival of CRC was significantly downregulated in this group (Fig. S5C). The positive control, regorafenib, a commonly used clinical drug for CRC treatment, was also in this group.

Additionally, we randomly selected ten out of these drug candidates to treat organoids at high, medium, and low concentrations respectively, and collected the drug-treated organoids for transcriptome sequencing analysis subsequently. The numbers of up and downregulated genes of each drug increased with drug concentrations, and the overlap part of differential expressed genes (DEGs) accounted for a large proportion of all the DEGs in the high and median concentration groups, indicating that the drug response signature is dose-dependent (Fig. S6).

Overall, we elucidated five representative drug response patterns based on the altered transcriptome profiles, and we further annotated these five patterns as differentiation induction group, growth inhibition group, metabolism inhibition group, immune response promoting group, and cell cycle inhibition group (Table S3). We also elaborately described the different characteristics of drug response signatures and the MOA of drug candidates that target CRC organoids, which provide clues for selecting potential drugs for clinical combination therapy.

### Validations of tumor suppression effects of drug candidates *in vivo*

**Figure 5.**
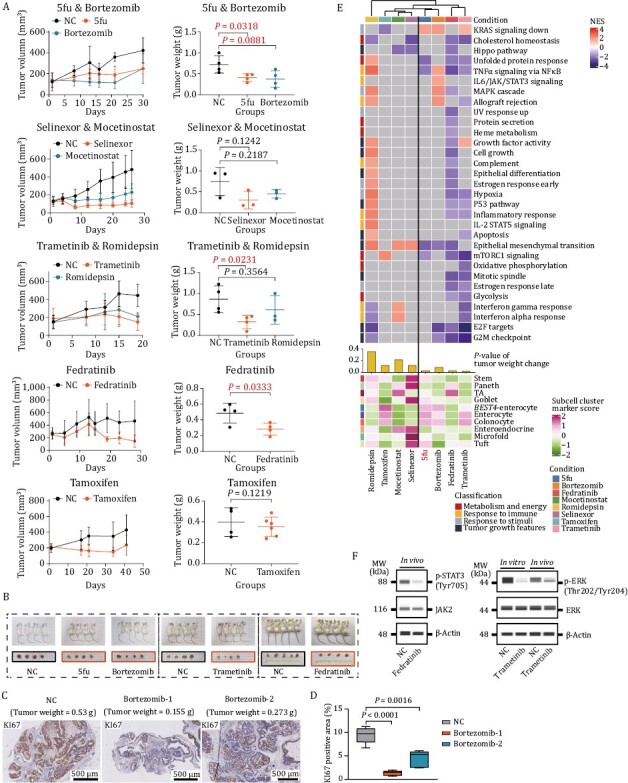
**Validation of anticancer effect of selected drugs on PDOX model.** (A) Anticancer effect of selected drugs. Fedratinib was administered at a concentration of 120 mg/kg twice daily (morning and evening) by oral gavage (4 mice). Bortezomib was administered at a concentration of 1 mg/kg twice weekly by intraperitoneal injection (4 mice). Tamoxifen was administered at a concentration of 50 mg/kg by oral gavage (6 mice). Trametinib was administrated at 1 mg/kg once daily by intraperitoneal injection (4 mice). 1.5 mg/kg romidepsin was administered twice weekly by intraperitoneal injection (3 mice). 20 mg/kg selinexor was administered three times a week by intraperitoneal injection (3 mice). Mocetinostat was administered at a concentration of 90 mg/kg by oral gavage (3 mice). Left plots showing tumor growth. Data are presented as mean ± SD. Right plots display xenograft tumor weight after treatment with vehicle versus selected drugs. *P*-values were determined by two-side *t*-test. (B) Images of mice and dissected tumors treated with different drugs. The side length of squares on the white paper background was 1 cm. (C) Representative images of KI67 staining in bortezomib-treated PDOX. Corresponding tumor weight was marked on the pictures. (D) Immunohistochemical analyses of KI67 expression in NC and bortezomib-treated PDOX. *P*-values were calculated by two-sided *t*-test. Bortezomib-1 and bortezomib-2 represent two different mice that treated with bortezomib. (E) Heatmap showing the expression levels of drug-disturbed tumor-associated signaling pathways and intestinal cell type marker genes of PDOX treated with eight drugs. (F) Capillary-based immunoassays of phospho-STAT3 and total JAK2 for organoids treated with fedratinib (2.5 μmol/L) for 96 h (left); capillary-based immunoassays of phospho- and total ERK for organoids treated with trametinib (0.1 μmol/L) for 96 h and mouse administrated with 1 mg/kg trametinib once daily (right).

To assess the performance of the drugs in the *in vivo* environments, PDOX models were successfully established in this study. By taking into consideration of the GR_50_ information and clinical toxicity of the drugs, one or two drugs with low GR_50_ values and low toxicity in clinical use were selected out from each drug response group (7 drugs in total) for tumor suppression experiments on PDOX. Besides, 5fu was used as a positive control. By counting the tumor weight in mice after drug treatment, we found that the median tumor masses were reduced after treatment with all of these eight drugs, indicating that consistent with the results of *in vitro* screening experiments, all the drugs we screened had potential tumor-suppressive effects. In addition to the commonly used clinical drug (5-fluorouracil, 44% reduction, *P*-value = 0.032), trametinib (41% reduction, *P*-value = 0.023), fedratinib (42% reduction, *P*-value = 0.033) also showed significant reduction in the tumor weight compared to the controls (Fig. 5A and 5B). Through immunoblotting experiment, we found that the p-ERK and p-STAT3 was reduced during treatment of trametinib and fedratinib, respectively, indicating that the activity of MAPK pathway was disturbed through trametinib treatment in CRC organoids, and fedratinib may inhibit CRC growth by inhibiting the JAK/STAT pathway (Figs. 5F and S7). Meanwhile, to further validate the anticancer effects of drug candidates, we conduct immunohistochemical (IHC) staining and found that the percentage of KI67 positive proliferating cells clearly decreased after the treatment by bortezomib (Fig. 5C and 5D). The above results suggest that the drugs we screened out might be promising candidates for clinical therapy of CRC patients.

Promoted by these results, we further validated the tumor suppression effects of the drugs at the transcriptome level. We dissected the tumors in mice after drug treatment and conducted RNA-seq. It was found that drugs were classified into two groups based on transcriptome characteristics. Drugs with better tumor-inhibition effects clustered together. In addition, trametinib, bortezomib, and fedratinib led to a significant downregulation of cell cycle-related pathway genes (Fig. 5E). Overall, by establishing the PDOX model *in vivo*, we found that all the drugs we screened out had the potential ability to inhibit CRC, validating the reliability of our drug screening system. In conclusion, transcriptomic data and protein level validation results complement each other and together confirmed the anticancer efficacy of drug candidates. And drugs with significant tumor suppression effects are likely to be useful in the treatment of CRC and may be used as a complement to the treatment regimen of current clinically used drugs.

### Comparison of the drug responses between CRC organoids *in vitro* and pairwise PDOX *in vivo*

**Figure 6.**
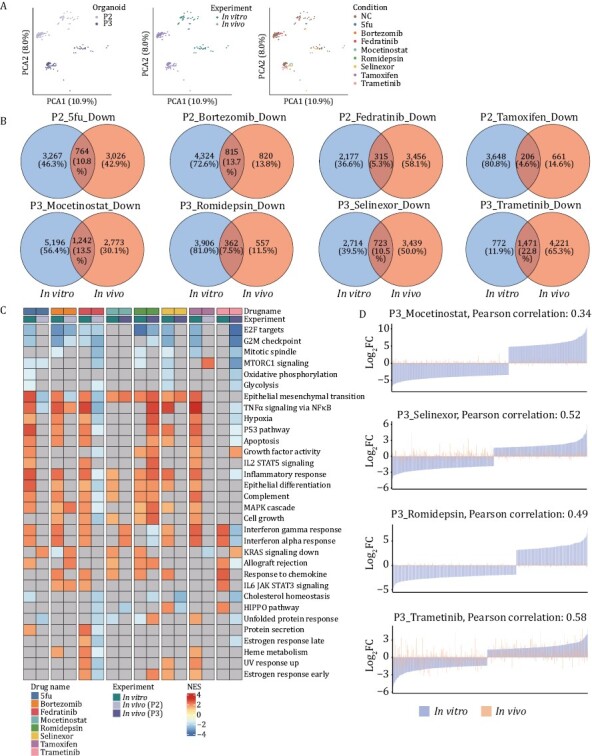
**Comparison of drug response transcriptome features of organoids cultured *in vitro* and PDOX established *in vivo*.** (A) PCA plots showing RNA-seq data of drug-treated organoids and PDOX. Patients’ information (left), experimental information (middle) and drug names (right) were projected onto the PCA plot. (B) Venn diagrams demonstrating the overlap of significantly downregulated genes between organoids and PDOX treated with different drugs. (C) Heatmap displaying the differences in the expression level of tumor-related pathways after drug treatment between organoids cultured and pairwise established PDOX. (D) Bar plots representing log_2_fold change values of top up-or down-regulated 250 genes expression in drug-treated samples vs. control samples, displaying the concordance and discordance of drug response signature between patient 3-derived organoids *in vitro* and paired PDOX *in vivo*.

Next, we further investigated the drug response differences between the *in vitro* and *in vivo* systems (organoid and pairwise PDOX models). To exclude the influence of the heterogeneities among patients, we compared the drug responses of PDOX and its corresponding organoids (Table S4). Inconsistent changes in the transcriptome of drug-treated cells *in vivo* and *in vitro* systems were observed, including the clinical-commonly used drug 5fu (Fig. 6A). We found that only approximately 10% of DEGs were overlapped between organoids *in vitro* and corresponding PDOX models *in vivo*, and the expression of the disturbed pathways differed between them (Figs. 6B, 6C and S8A). Meanwhile, the concordance expression of cancer-related pathways between these two screening systems varied among different organoid lines and different drug treatments (Fig. S8B). We speculate that it might be caused by the underdose of the drug treatment *in vivo*, which rendered the modulation of the signaling pathways and expression of the genes. Since the size of the tumors in mice is much larger than that of the *in vitro* cultured organoids, the extent of penetration of drug molecules into the tumors may be relatively reduced. To test this hypothesis, we extracted the top 250 genes strongly upregulated or downregulated in both CRC organoids and pairwise PDOX models. We found that in one of the organoid lines, the correlation coefficient of gene expression was on average of 0.48 (Fig. 6D). However, we did not get the same result in the other organoid line (Fig. S8C), indicating that the underdose could only partially explain the divergent transcriptomic features between organoids and pairwise PDOX models, and whether the inherent biological differences between these two systems (such as the exposure of oxygen, presence of vessels and the recruitment of microenvironment cells) influence the gene expression changes need to be investigated further.

### Organoid system combining with drug prediction could help discovering potential drug combinations

**Figure 7.**
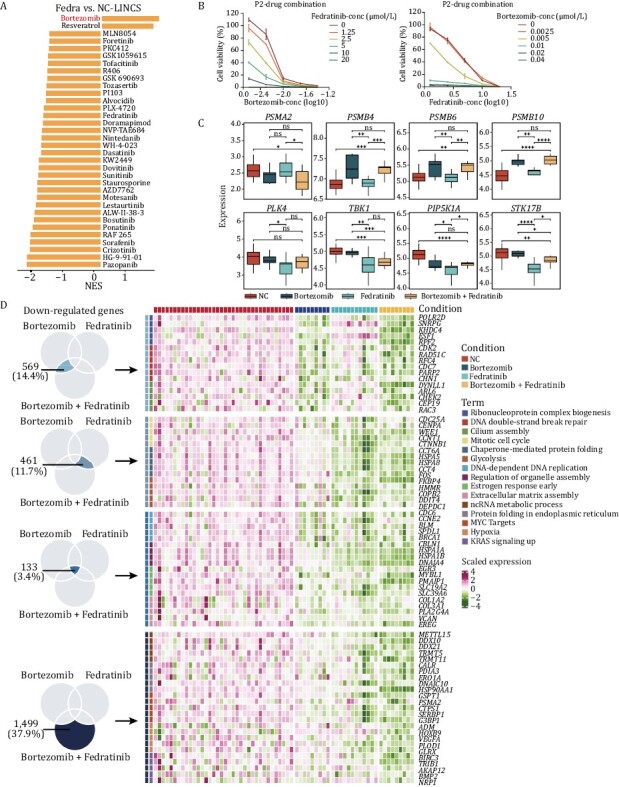
**Exploring the feasibility of organoids as model for discovering drug combination.** (A) Prediction of drugs that may have synergistic effects with fedratinib. Bortezomib and resveratrol that had negative NES values were considered candidate drugs for combination with fedratinib. (B) Cell viability of patient 2-derived organoids exposed to the combination of fedratinib and bortezomib. Concentration of fedratinib were set as 20 μmol/L, 10 μmol/L, 5 μmol/L, 2.5 μmol/L, 1.25 μmol/L. Concentration of bortezomib were set as 0.04 μmol/L, 0.02 μmol/L, 0.01 μmol/L, 0.005 μmol/L, 0.0025 μmol/L. Each point representing values collected from two replicates, and the error bar representing mean ± SD. (C) Expression level of the targets of bortezomib (*PSMA2*, *PSMB4*, *PSMB6*, and *PSMB10*), and the downstream targets of fedratinib (*PLK4*, *TBK1*, *PIP5K1A*, *STK17B*). *P* values were determined by two-side *t*-test. ^*^P < 0.05, ^**^P < 0.01, ^***^P < 0.001, ^****^P < 0.0001. (D) Heatmap showing the expression level of significantly downregulated genes and their corresponding pathways in different groups.

Next, we want to further investigate whether the drug prediction approach and organoid-based drug screening system could be used to obtain valuable clues for the drug combination treatment for CRC (Fig. S8D). Among drugs with potential tumor suppression effect, we found that fedratinib is of great interest. Fedratinib was initially developed for the treatment of myelofibrosis (Mullally et al., 2020). In our study, Fedratinib showed significant inhibition of CRC tumor organoids. Thus, we utilized the LINCS database to predict the drugs that may have synergistic effects with fedratinib. We obtained two drugs that combined with fedratinib which may have better tumor suppression effects. Bortezomib was the top-ranked drug candidate (Fig. 7A). To test the effectiveness of the drug combination, we first set gradient concentration of the combination of the drugs based on two patient-derived organoid lines. The tumor suppression effect of the combination group was greater than those of individual drugs, indicating that the combination of fedratinib and bortezomib might have a greater tumor-inhibition effect (Figs. 7B and S8E).

We further verified the synergistic effects in the PDOX model. Since the combination of the drugs was toxic to mice, so we used the half dose for each drug. Although the significant suppression effect was not obtained when we reduced drugs’ concentration (Fig. S8F), we verified the synergistic effects at the transcriptomic level. The tumors were dissected from the mice after the drug treatment and were used for transcriptome sequencing analysis. Proteasome subunits such as *PSMA2*, *PMSB4*, *PMSB6*, and *PMSB10* were identified as the targets of bortezomib, and *PLK4*, *TBK1*, *RIP5K1A*, and *STK17B* were the downstream targets of fedratinib. In the combination group, both groups of these drug target genes were significantly downregulated (Fig. 7C). More importantly, among the DEGs after drug treatment, we identified key genes that participated in cancer-related pathways in both the single drug treatment group and the combination treatment group (Fig. 7D). Combining with transcriptome data and experimental validations both *in vitro* and *in vivo*, we observed the synergistic effect of our predicted drug combination group, indicating that organoid model system combining with drug prediction could help discovering potential combination drug treatment.

## Discussion

As a valuable alternative to traditional monolayer cultures and PDX models, the organoid systems have shown clear advantages in being more cost-effective and better reproducing the characteristics of *in vivo* tumor tissues. It has been widely reported that patient-derived organoids have been proved well predict the drug responses of cancer patients, indicating that the organoid model has great values for applications in drug discovery (Vlachogiannis et al., 2018; Yao et al., 2020). However, the MOA of drugs based on organoid culture system have not been well described, and there is a great need conducting individualized drug screening. Here, we successfully established a CRC organoid-based drug screening system. To increase the likelihood of screening out promising therapeutic drug candidates for CRC, we combined computational drug prediction with experimental validations both *in vitro* and *in vivo*. More importantly, by performing transcriptomic analysis, we have provided valuable clues to dissect the MOA of drug candidates targeting CRC.

With transcriptome and genome data, we first evaluated the molecular characteristics of the established CRC tumor organoids. We revealed that tumor organoids could well preserve both the morphological features and molecular features of the patients’ tumor tissues in situ. Notably, as previously reported, the gene mutations could be well preserved even after long-term culture and expansion *in vitro*, which is imperative for subsequent interpretations of drug response mechanisms. Moreover, organoid biobank covered different mutation subtypes, and its application in drug screening could yield key associations between mutation types and drug sensitivities, such as CRC organoids harbored *TP53* mutation was insensitive to MDM2 inhibitors (van de Wetering et al., 2015), the missense mutations of *ARID1A* in pancreatic cancer are associated with increased sensitivity to dasatinib (Hirt et al., 2022), which showed a great potential for finding drugs targeting specific type of patients. Here, we demonstrate the comprehensive mutational patterns of our established organoid lines, and the distinct mutation types of tumor organoids enable us to observe heterogeneous drug responses among different lines of CRC organoids. However, due to the large heterogeneities of CRC and limited sample numbers, it prevented us from drawing important patterns and conclusions on the correlations between gene mutations and drug sensitivities. In the future, a larger panel of organoids with various types of mutations and organoids derived from different tumor sites of same patient are required for further investigations.

Drug sensitivity databases are gradually serving as valuable resources for facilitating the discovery of anticancer drugs (Seashore-Ludlow et al., 2015; Subramanian et al., 2017). In this study, we conducted a ridge regression-based strategy to predict potential drugs for CRC clinical therapies. By incorporating disease signatures and drug-response signatures, we obtained five drug candidates, three of which have been further validated having potential tumor suppression effects. Meanwhile, we presented an organoid-based screening platform and conducted a repurposing drug screening. A total of 335 drugs that have already passed phase I clinical trial for at least one human disease were screened. Since the safety of the screened drugs has been well-validated, the risk of adverse effects was largely reduced. In addition, PDOX that we successfully established provided validations of our screening results *in vivo*. Although the drugs we screened out by organoid-based screening system were previously approved for the treatment of tumor- or non-tumor diseases (Voorhees et al., 2003; Mullally et al., 2020), they exhibited potential CRC tumor-suppressing effects. The above results suggested that computation-based prediction approaches and experiment-based approaches could complement each other, improving the screening success rate. Comparing with current drug screening methods which only conduct screening on the experiment level, our established strategy combined with computation-based drug prediction to narrow down the target screening drugs, and provided a novel way to discover potential drugs more efficiently.

More importantly, we provided a transcriptome profile of organoids treated with potential drug candidates and we further divided the drug response signatures into five representative patterns (differentiation induction group, growth inhibition group, metabolism inhibition group, immune response promoting group, and cell cycle inhibition group), which provided a better understanding of the MOA of the drugs. We demonstrated that drugs in different groups exhibited distinct response signatures. For example, organoids treated with drugs in Pattern 1 (differentiation induction group) showed an upregulation of the expression of genes that participated in cell differentiation. It has been reported that the frequency of stem-like cells was associated with the maintenance and progression of tumors (Fumagalli et al., 2020), inhibiting which may resulting in tumor regression (Ordonez-Moran et al., 2015). So, we assumed that Pattern 1 drugs could suppress the growth through induce the differentiation of the tumor cells. Interestingly, we yielded an immune-related Pattern 4 (immune response promoting group), drugs in which group could both suppress the growth and promote the antigen presentation effects of the tumor cells, which may enhance the recruitment of immune cells to attack these tumor cells. We found that this group of drugs is of great interest. Increasing evidences suggested that modulating immune microenvironment could help increasing cellular responses to immunotherapy (Yap et al., 2021). For example, MEK inhibitors could display both anticancer activities and promotion of antigen presentation activities, which could potentially enhance the immune therapeutic effects (Limagne et al., 2022). We assumed that the drugs we screened out in Pattern 4 are potentially great candidates for combinational immunotherapy. Due to the relatively high number of drug candidates we analyzed, we speculated that these five identified patterns represent general responses of CRCs to small-molecule drugs. To our knowledge, our study is the first to describe a drug response transcriptome analysis based on CRC patients-derived organoid culture system, providing a sequencing-based strategy for drug discovery and provides clues to the selection of potential drugs for clinical combination therapy. Further integrated transcriptomic analysis with clinical data could accelerate the discovery of drugs that target specific subtypes of CRC or help to find inhibitors that can overcome the resistance of chemotherapeutic drugs.

Furthermore, previous studies have shown that xenografted tumors undergo mouse-specific evolution under the *in vivo* mouse environment, resulting in altered characteristics of the tumor itself (Ben-David et al., 2017). In this study, discordant transcriptome profiles of *in vitro* and *in vivo* tumor cells after drug treatment were captured, including the first-line clinical drug 5fu, which also displayed diverse mechanisms on these two screening systems. By comparing the expression of top variable genes *in vitro* and *in vivo*, we found that the influence of drug dose could only partially explain the differences between them. Therefore, whether mouse microenvironment-specific tumor evolution occurs in our organoid-based xenograft system and whether organoids perform better on exploring the underline mechanism of drugs required further exploration.

Overall, we constructed a drug repurposing screening system for the discovery of anticancer drugs and screened out 34 drugs that could be potential therapeutic drug candidates for CRC, highlighting the value of our computation-based screening approach. More importantly, integrating with transcriptome sequencing, the mechanism of action of drug candidates was successfully revealed. Meanwhile, the resulting transcriptome profiles serve as a valuable resource for the repurposed drug discovery.

## Materials and methods

### Establishment of patient-derived tumor organoids

CRC specimens were provided by the Peking University Third Hospital. Fresh tumor samples were stored in the antibiotic-containing DMEM medium with 10% fetal bovine serum (C04001-500, VivaCell) after surgically resected and transported to the laboratory at 4°C for immediate processing. The establishment and culture of colorectal tumor organoids was performed as previously described (Wang et al., 2022). Briefly, after being washed gently at least five times with pre-chilled 1X DPBS, tumor tissues were cut into small pieces using surgical scissors and digested with 2.5 mg/mL type II and type IV collagenase (17101015 and 17104019, Invitrogen) to obtain single-cell suspensions. After digestion, the suspension was passed through a 40 μm cell strainer to remove undigested parts, and then centrifuged at 800 ×*g* for 5 min. The pellet was resuspended with Matrigel (356231, Corning) and dispensed into a 24-well cell culture dish. After 30 min of solidification of Matrigel, conditioned medium was then added. Conditioned medium was prepared according to the previously reported protocol (Miyoshi and Stappenbeck, 2013). At early passages, 10 μmol/L ROCK inhibitor Y-27632 (S6390, Selleck) was supplemented to the medium.

### Preparation of pharmaceutical compounds

All 335 pharmaceutical compounds utilized for the *in vitro* screening were purchased from Selleck. The drugs were stored at −80°C and repeated freezing and thawing were avoided. For *in vitro* screening assay, the concentration of the drugs used in the primary screening was 10 μmol/L. And six-point dose dilution series was set for the secondary screening. To get better interpretable value, drugs were diluted at 3.16 (half-log) times, 2 times, 5 times or 10 times respectively, depending on the sensitivity of the organoids’ response to the drugs. Three replicates were set up for each drug treatment, and 50 μL of the diluted drug was added to each well.

As for *in vivo* assay, drugs were diluted in different ways according to their characteristics. Dilution of fedratinib (S2736, Selleck): Fedratinib was administered at a concentration of 120 mg/kg twice daily (morning and evening) by oral gavage (Geron et al., 2008). 100 mg powder of fedratinib was dissolved with 0.16 mL of DMSO to make a concentrated reservoir of 625 mg/mL and was thoroughly mixed and dissolved at 37°C. Fedratinib storage was further diluted with corn oil (405435000, Acros) and prepared for administration. Dilution of bortezomib (S1013, Selleck): Bortezomib was administered at a concentration of 1 mg/kg twice weekly by intraperitoneal injection (Stewart et al., 2017). 25 mg of drug powder was dissolved with 1 mL of DMSO, which was diluted 100-fold with 0.9% saline to obtain a working solution of 0.25 mg/mL. Dilution of tamoxifen (T5648, Sigma-Aldrich): Tamoxifen was administered at a concentration of 50 mg/kg by oral gavage. 100 mg of tamoxifen was dissolved with 10 mL of corn oil. After incubated in a rotating incubator at 37°C, tamoxifen was well dissolved for about 1 h. Dilution of trametinib (S2673, Selleck). Trametinib was administrated at 1 mg/kg once daily by intraperitoneal injection (Dizdar et al., 2019). 10 mg of trametinib was dissolved with 0.5 mL of DMSO, which was then diluted 100-fold with corn oil for administration. Dilution of romidepsin (S3020, Selleck): 1.5 mg/kg drug was administered twice weekly by intraperitoneal injection (Jiang et al., 2020). 10 mg of powder was dissolved with 333 μL of DMSO to obtain 30 mg/mL of concentrated stock, which was then diluted with 0.9% saline to obtain 0.3 mg/mL of working solution. Dilution of selinexor (S7252, Selleck): 20 mg/kg drug was administered three times a week by intraperitoneal injection (Etchin et al., 2013). 50 mg of drug was resuspended with 0.5 mL to make a 100 mg/mL concentrate, and was then diluted 25-fold in 0.9% saline. Dilution of mocetinostat (S1122, Selleck): administered at a concentration of 90 mg/kg by oral gavage (Bonfils et al., 2008). A working solution of 18 mg/mL was obtained by adding 0.111 mL of DMSO to 50 mg of powder, diluting 25-fold with corn oil and administering by gavage to mice after resuspension in corn oil.

### ATP-based cell viability assay

A total of eight organoids were used for drug screening. The procedure for drug screening was as follows: tumor organoids were first digested with TrypLE (12604021, Gibco) for 5–8 min at 37°C. After confirming that most of the cells were digested into single cells under a microscope, we collected the cells by centrifugation at 800 ×*g* for 5 min. The supernatant was further removed and the cells were suspended in 1 mL of culture medium. Cells were centrifugated after being filtered through a 70 μm filter and then resuspended with BME (3533-010-02, R&D Systems). Subsequently, single-cell suspension was dispensed into 96-well plate (approximately 3000 cells per well). After 10 min of solidification in the incubator at 37°C, 50 μL of the medium was added to each well. 48 h later, the medium that contained diluted drugs was replaced and the ATP values of the cells in three wells were measured as the initial values for calculating GR_50_. 0.1% DMSO was used as the negative control. To avoid evaporation, 200 μL of sterile water was added to the surrounding wells. After 120 h of drug treatment, ATP values were examined using CellTiter-Glo 3D (G9683, Promega) reagent according to the manufacturer’s instruction. In the primary screening, the ratio of organoids treated with drugs compared to negative control was under 0.3 were considered significantly inhibit CRC organoids. ATP values at the start of the drug treatment and ATP values at the end of the drugs treatment were collected for GR value calculation. GR_50_ was finally calculated using the R package GR metrics.

### Organoid-based xenotransplantation

Patient 2- and patient 3-derived organoids were harvested using cell recovery solution (354253, Corning), which could completely dissolve BME while keeping organoids intact. After centrifugation at 400 ×*g* for 5 min, organoids were resuspended with 5 mL of DPBS. After thorough mixing, 50 μL of the organoid was then taken from 5 mL and proceeded to digestion into single cells with TrypLE for cell counting. Depending on the number of mice, a certain number of organoids were taken and resuspended in 50% Matrigel. 100 μL of the mixture was injected into the subcutaneous of the right forelimb of 4-week-old NOD/SCID mice with a 1 mL sterile syringe (3–4 million cells per mice). The rate of tumor formation differed from organoids and we routinely monitored the tumor size. When the tumor grew to 150–200 mm^3^, the mice were randomly grouped and the drug treatment start. The mice were weighed routinely, and the length and width of the tumors were recorded to calculate the tumor volume (tumor volume = length × width × width/2). When the tumor diameter exceeded 20 mm or significant weight loss occurred, the experiment was stopped and the mice were put to death. Mice were euthanized after a maximum of 40 d of treatment. The tumors that were dissected from mice were placed in Advanced DMEM/F12 containing 10% FBS and then divided into small pieces with scissors. Some of the pieces were put into RNA*later* (AM7021, Invitrogen) solution for storage and the subsequent total RNA acquisition. The remaining pieces were placed in 4% PFA (P110, Macgene) for fixation and prepared for the IHC staining.

### Capillary-based immunoassay

Organoids treated with fedratinib (2.5 μmol/L) or trametinib (0.01 μmol/L) for 96 h were collected by TrypLE. As for PDOX, tumor that resected from mice after drug treatment were grinded and prepared for protein extraction. Cell lysis buffer contained protease inhibitor and phosphatase inhibitor were used to extracted protein. Protein separation and detection were performed on Simple Western system and Compass software (Protein Simple). Antibodies against the following proteins were used: ERK1/2 (#4695, CST, 1:50 diluted), phosphor-ERK1/2 (T202/Y204) (#4370, CST, 1:50 diluted), JAK2 (#3230, CST, 1:20 diluted), phosphor-STAT3 (Tyr705) (#9145, CST, 1:20 diluted), and β-actin (#4970, CST, 1:50). HRP-conjugated secondary antibody was used to detect the signals and results were visualized using proteinsimple software.

### Immunohistochemical staining

Tumor tissues resected from mice were first fixed with 10% neutral buffered formalin and further embedded in paraffin. Then, the embedded blocks were sectioned into 8-μm-thick slices. For antigen retrieval, slices were soaked in 0.01 mol/L citrate buffer and boiled for 30 min. After treatment with 3% hydrogen peroxide solution, the slices were blocked with 10% BSA at room temperature for 1 h. Primary antibody (KI67, ab15580, diluted in 1:200; p-JAK2, CST3776, diluted in 1:100) was then diluted and added to the slice. After incubation at 4°C overnight, the secondary antibody was added and then incubated at room temperature for 30 min. DAB substrate liquid was finally added to visualize the slices. The positive cell ratios of three random-picked pictures were calculated respectively with ImageJ 1.47v software. *P*-values were calculated by two-sided *t*-test. ^*^P < 0.05. ^**^P < 0.01.

### Bulk transcriptome sequencing

Total RNA was extracted using the RNeasy Mini Kit (74104, Qiagen). For the drug-treated organoids: four organoids were collected after 120 h of drug treatment and proceed to RNA extraction respectively. To avoid cell loss and mechanical damage to the cells during collection of the organoids, we used cell recovery solution (354253, Corning) to digest the Matrigel which preserving the integrity of the organoids. For the tumor tissues: tumor tissues that resected from the mice were stored in RNA*later* solution (AM7021, Invitrogen). QIAshredder (79656, Qiagen) was used to filter the undigested clumps. After RNA extraction, mRNA was isolated and amplified according to the instructions of the NEBNext® Ultra™ II RNA Library Prep Kit (E7770L, NEB). Afterwards, DNA was quantified by Equalbit 1X dsDNA HS Assay Kit (EQ121-01, Vazyme). Approximately 50 ng of amplified cDNA was used to perform library construction following the instructions of the KAPA Hyper Prep Kit (KK8054, KAPA).

### Processing of bulk whole genome sequencing and whole exome sequencing data

For the WGS and whole exome sequencing (WES) data, we used fastp (version 0.23.1) to trim reads of low quality or with adaptors and used BWA (version 0.7.17-r1188) to map reads to the hg38 reference genome. As for the WGS data, Control-FRCC (version 11.6) was used to call CNV with the parameter ploidy set to 2. As for the WES data, the GATK (version 4.0.12) was used to call germline mutations following the manual of GATK. The Haplotype mode was used to identity germline mutations and reference SNV resources including hapmap_3.3, 1000G omni_2.5, 1000G phase1 snps, dbsnp138, mills and 1000G gold standard indels were used to filter germline mutations. Only “PASS” germline mutations with high confidence were retained and the transformed maf files were used for further analysis.

### Processing of bulk whole genome bisulfite sequencing data

Fastp (version 0.23.1) was used to trim and filter sequencing reads. Bismark (version 0.23.1) was used to map whole genome bisulfite sequencing (WGBS) data to the hg38 reference genome. The methylated C ratio of sites were also calculated and extracted by bismark. We used the R package methylKit (version 1.10.0) to identify differential methylated sites and differential methylated regions (DMR), with the differential methylation cutoff set to 0.25, *q*-value set to 0.01 and window length of DMRs set to 500 bp.

### Processing of bulk RNA-seq data

We used fastp (version 0.23.1) to trim reads of low quality or with adaptors and used STAR (version 2.7.0f) to map reads to hg38 reference genome. FeatureCounts (version 2.0.1) was used to calculate counts of every gene. The R package DESeq2 (version 1.24.0) was used to identify DEGs, and the gene count matrix was inputted. The adjusted *P*-value cutoff was 0.05, and genes with log2 of transcript per million (TPM) more than 1 were retained as DEGs of different conditions. The log2 of fold change were not considered to involve more DEGs in conditions. The principal component analysis (PCA) was performed by R packages factoextra (version 1.0.5) and FactoMineR (version 1.42).

### Gene set enrichment analysis

The GO analysis was performed by clusterProfiler (version 3.18.0) or the online tool Metascape (Zhou et al., 2019). As for the GSEA analysis, the GSEA function of clusterProfiler (version 3.18.0) was used to perform the GSEA analysis (Yu et al., 2012). The hallmark gene sets and ontology gene sets were involved in GSEA analysis. And we used the GSVA (Gene Set Variation Analysis) enrichment scores to describe enrichment scores of gene sets and signatures through the R package GSVA (version 1.32.0) (Hanzelmann et al., 2013). The “ssgsea” method of GSVA package was used. Related pathways or signatures were summarized from the KEGG database.

### Comparison drug effects *in vitro* and *in vivo* based on correlations of gene sets

As for the selected gene sets or signatures, we calculated the Pearson correlation coefficient of the sample conditions *in vitro* and *in vivo*. The correlation of *in vitro* and *in vivo* was used to assess the similarity based on the specific gene set or signature, which indicates the consistency of the drug.

### The survival analysis

Survival analysis of CRC samples from the TCGA dataset based on the expression status of identified genes was carried out by the survival package (version 0.4.8) and the survminer package (version 2.44-1). The assumption of the Cox proportional hazards model was tested using the Cox with 0.1 as the cutoff value, and the Cox proportional hazards model was fit using patient groups divided by the median gene expressing level.

### Prediction of candidate drugs and targets of CRC for drug combinations

We mainly used a ridge regression-based method of the R package pRRophetic (version 0.5) to predict the IC_50_ AUC values of potential drug responses for CRC (Geeleher et al., 2014). The transcriptomic data of cell lines from CCLE and drug response data from CTRP, PRISM, GDSC1 and GDSC2 were utilized as training sets, which contains about 2,521 drugs and 1,969 cell lines. The gene expression dataset of CRC samples from a scRNA-seq data set (GSE144735) with about 6,226 epithelial cells was used as the prediction dataset. And the predicted IC_50_ AUC values of involved drug candidates were as the predicted results through the calcPhenotype function. Pseudo-tumor and normal samples were obtained by averaging of gene expression from single cells. The gene set associated with poor prognosis of CRC samples in TCGA dataset was also identified with the hazard ratio less than 0.6 and the adjusted *P* value less than 0.05. Three criteria were used to filter and identify the drug candidates of CRC. First, drugs that the log2 of fold change of the predicted IC_50_ AUC values between tumor and normal samples is less than −0.1, and the adjusted *P* values is less than 0.05 (under the *t*-test) could be retained. Second, drugs that the Pearson correlation of the predicted IC_50_ AUC value and the poor prognosis associated gene set score is less than −0.5 could be retained. Third, the tumor gene set score was calculated according to the used scRNA-seq dataset. Drugs that the Pearson correlation of the predicted IC_50_ AUC value and the tumor gene set score is less than −0.3 could be retained. If all the three criteria were achieved, these drugs were retained as drug candidates of CRC.

As for the prediction of drug combination, we identified DEGs with their log2 of fold change of fedratinib and negative control. The GSEA method was used and drug binding gene sets of LINCS were used as inputs (Musa et al., 2019). In details, upregulated genes of fedratinib were viewed as the target of combination drug theoretically, and gene sets associated with drugs in which upregulated signatures of fedratinib enriched were viewed as drug candidates for combination with fedratinib.

### The similarity of drug responses *in vitro* and *in vivo*

The log2 of fold change values of drug-treated samples and control samples *in vitro* and *in vivo* were used to evaluate the similarity. Pearson correlation of the mean values of the log2 of fold changes *in vitro* and *in vivo* was calculated as the assessment of the similarity of drug responses *in vitro* and *in vivo*.

## Supplementary information

The online version contains supplementary material available at https://doi.org/10.1093/procel/pwad038.

pwad038_suppl_Supplementary_Figures

pwad038_suppl_Supplementary_Table_S1

pwad038_suppl_Supplementary_Table_S2

pwad038_suppl_Supplementary_Table_S3

pwad038_suppl_Supplementary_Table_S4

## Data Availability

The raw sequence data reported in this paper have been deposited in the Genome Sequence Archive (GSA) in National Genomics Data Center (National Genomics Data Center Members and Partners, 2020), Beijing Institute of Genomics (China National Center for Bioinformation), Chinese Academy of Sciences, under accession number HRA002507.

## Abbreviations

CRC: colorectal cancer
CNV: copy number variation
DEG: differentially expressed gene
GSEA: gene set enrichment analysis
MOA: mechanisms of action
PDOX: patient-derived organoid-based mouse xenograft
SNV: single nucleotide variation
WES: whole-exome sequencing

## Code availability

All data analyses were followed by standard programs and codes are available on reasonable request.
