# Supplementary material for: Drug repurposing screening and mechanism analysis based on human colorectal cancer organoids
Source: Protein Cell. 2023 Jun 22;15(4):285–304. doi: 10.1093/procel/pwad038 (PMC10984622; doi:10.1093/procel/pwad038)
Supplement: pwad038_suppl_Supplementary_Figures [file pwad038_suppl_supplementary_figures.pdf]

Fig. S1

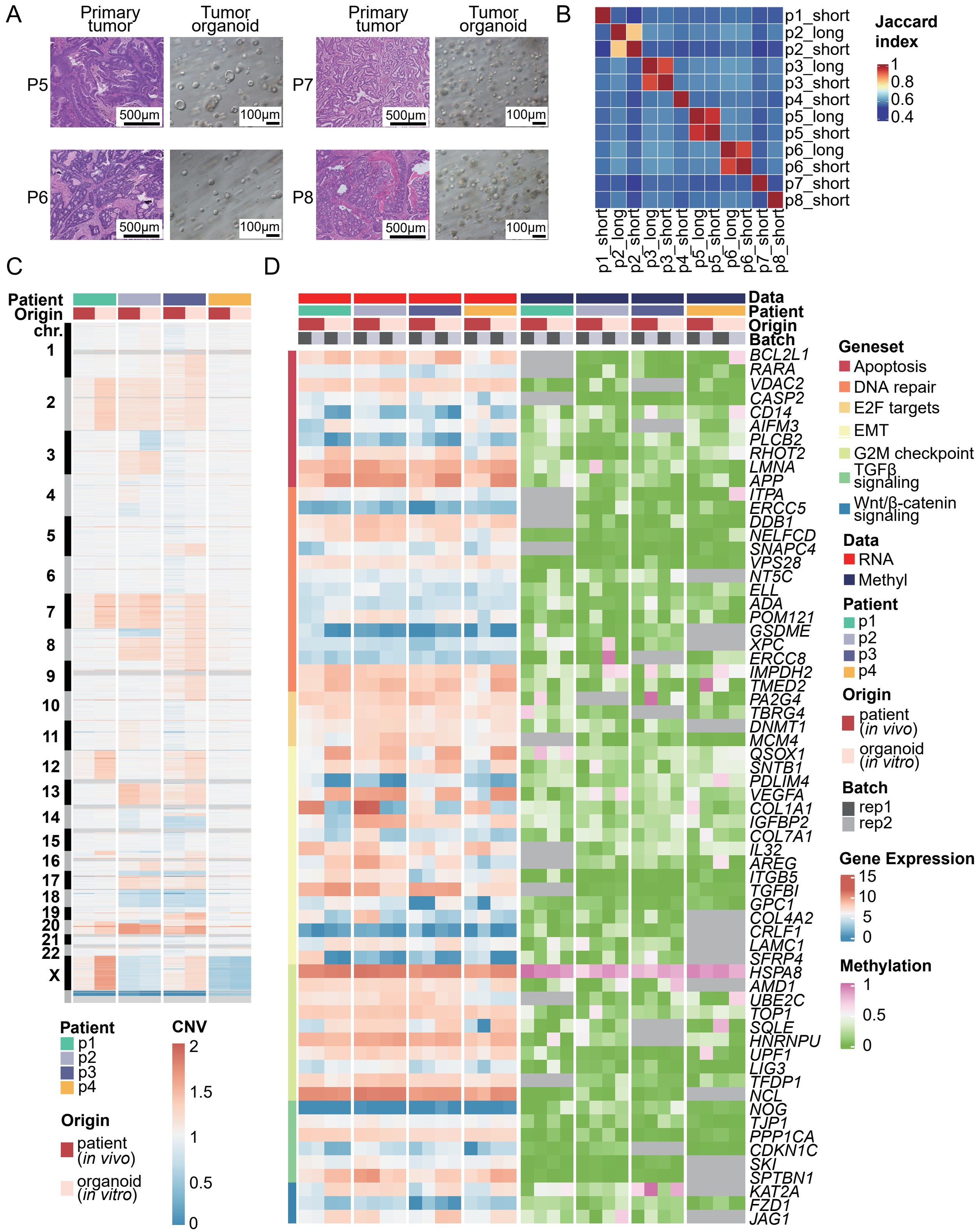
A
Primary tumor
Tumor organoid
P5
P6
P7
P8
500μm
100μm
B
Jaccard index
1
0.8
0.6
0.4
p1_short
p2_long
p2_short
p3_long
p3_short
p4_short
p5_long
p5_short
p6_long
p6_short
p7_short
p8_short
C
Patient
Origin
chr.
1
2
3
4
5
6
7
8
9
10
11
12
13
14
15
16
17
18
19
20
21
22
X
Patient
p1
p2
p3
p4
Origin
patient (in vivo)
organoid (in vitro)
CNV
2
1.5
1
0.5
0
D
Data
Patient
Origin
Batch
BCL2L1
RARA
VDAC2
CASP2
CD14
AIFM3
PLCB2
RHOT2
LMNA
APP
ITPA
ERCC5
DDB1
NELFCD
SNAPC4
VPS28
NT5C
ELL
ADA
POM121
GSDME
XPC
ERCC8
IMPDH2
TMED2
PA2G4
TBRG4
DNMT1
MCM4
QSOX1
SNTB1
PDLIM4
VEGFA
COL1A1
IGFBP2
COL7A1
IL32
AREG
ITGB5
TGFBI
GPC1
COL4A2
CRLF1
LAMC1
SFRP4
HSPA8
AMD1
UBE2C
TOP1
SQLE
HNRNPU
UPF1
LIG3
TFDP1
NCL
NOG
TJP1
PPP1CA
CDKN1C
SKI
SPTBN1
KAT2A
FZD1
JAG1
Geneset
Apoptosis
DNA repair
E2F targets
EMT
G2M checkpoint
TGFβ signaling
Wnt/β-catenin signaling
Data
RNA
Methyl
Patient
p1
p2
p3
p4
Origin
patient (in vivo)
organoid (in vitro)
Batch
rep1
rep2
Gene Expression
15
10
5
0
Methylation
1
0.5
0

Fig. S2

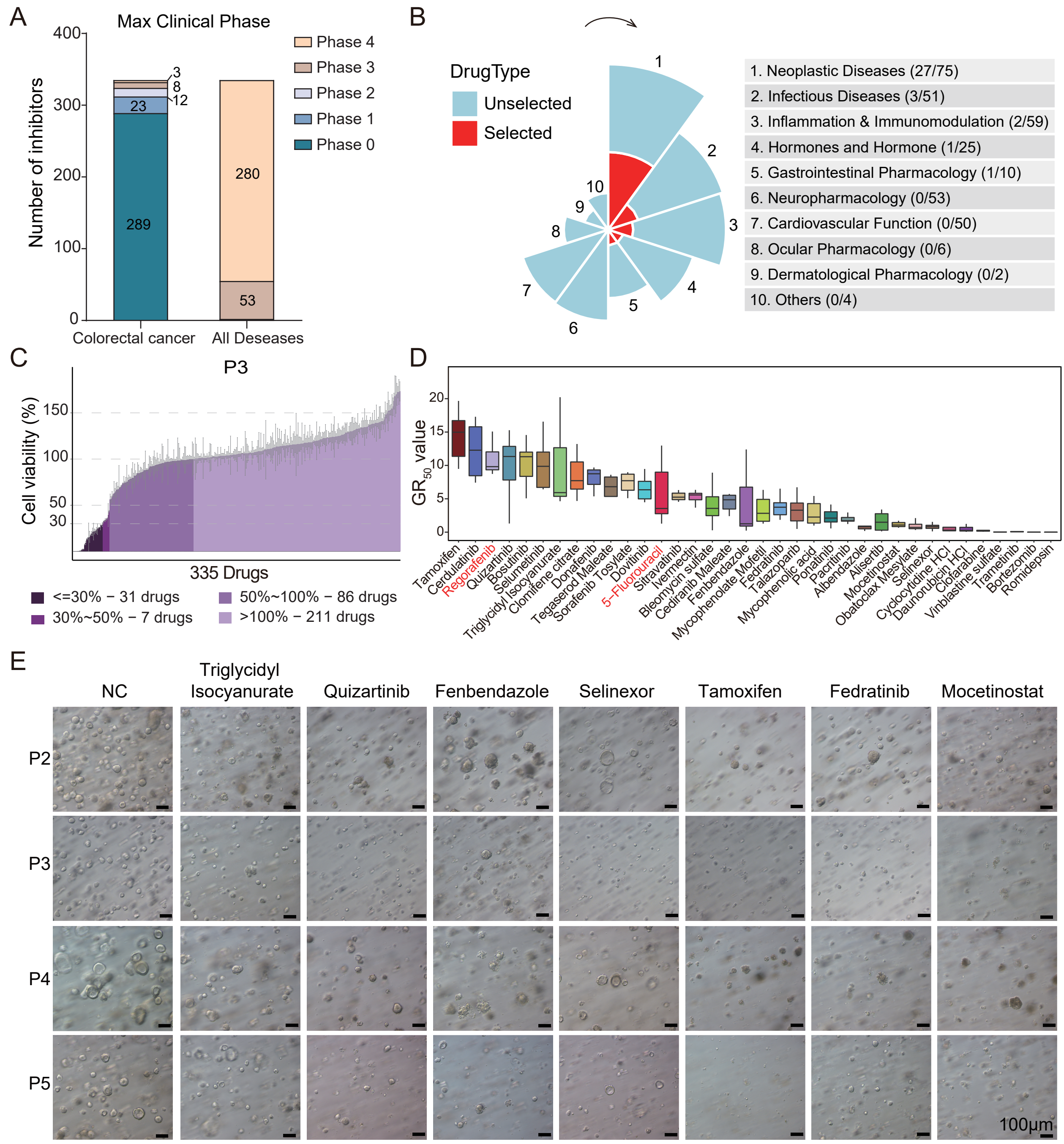
A
Max Clinical Phase
Number of inhibitors
400
300
200
100
0
Phase 4
Phase 3
Phase 2
Phase 1
Phase 0
3
8
12
23
289
280
53
Colorectal cancer
All Deseases
B
DrugType
Unselected
Selected
1
2
3
4
5
6
7
8
9
10
1. Neoplastic Diseases (27/75)
2. Infectious Diseases (3/51)
3. Inflammation & Immunomodulation (2/59)
4. Hormones and Hormone (1/25)
5. Gastrointestinal Pharmacology (1/10)
6. Neuropharmacology (0/53)
7. Cardiovascular Function (0/50)
8. Ocular Pharmacology (0/6)
9. Dermatological Pharmacology (0/2)
10. Others (0/4)
C
P3
Cell viability (%)
150
100
50
30
335 Drugs
<=30% − 31 drugs
30%~50% − 7 drugs
50%~100% − 86 drugs
>100% − 211 drugs
D
GR_{50} value
20
15
10
5
0
Tamoxifen
Cerdulatinib
Regorafenib
Quizartinib
Bosutinib
Selumetinib
Triglycidyl Isocyanurate
Clomifene citrate
Donafenib
Tegaserod Maleate
Sorafenib Tosylate
Dovitinib
5−Fluorouracil
Sitravatinib
Ivermectin
Bleomycin sulfate
Cediranib Maleate
Fenbendazole
Mycophenolate Mofetil
Fedratinib
Talazoparib
Mycophenolic acid
Ponatinib
Pacritinib
Albendazole
Alisertib
Mocetinostat
Obatoclax Mesylate
Selinexor
Cyclocytidine HCl
Daunorubicin HCl
Clofarabine
Vinblastine sulfate
Trametinib
Bortezomib
Romidepsin
E
NC
Triglycidyl Isocyanurate
Quizartinib
Fenbendazole
Selinexor
Tamoxifen
Fedratinib
Mocetinostat
P2
P3
P4
P5
100μm

Fig. S3

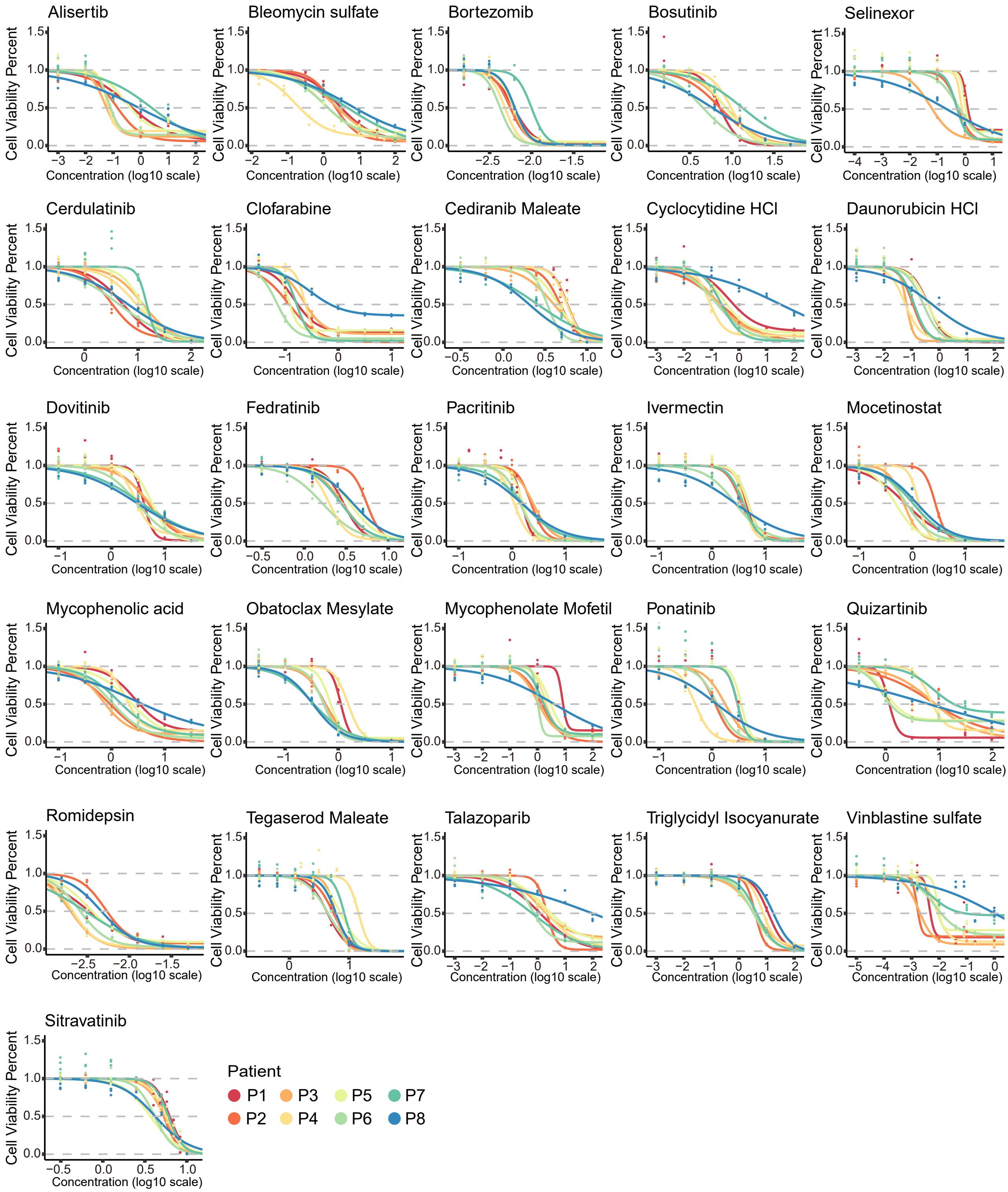
Alisertib
Bleomycin sulfate
Bortezomib
Bosutinib
Selinexor
Cerdulatinib
Clofarabine
Cediranib Maleate
Cyclocytidine HCl
Daunorubicin HCl
Dovitinib
Fedratinib
Pacritinib
Ivermectin
Mocetinostat
Mycophenolic acid
Obatoclax Mesylate
Mycophenolate Mofetil
Ponatinib
Quizartinib
Romidepsin
Tegaserod Maleate
Talazoparib
Triglycidyl Isocyanurate
Vinblastine sulfate
Sitravatinib
Cell Viability Percent
Concentration (log10 scale)
Patient
P1
P2
P3
P4
P5
P6
P7
P8

Fig. S4

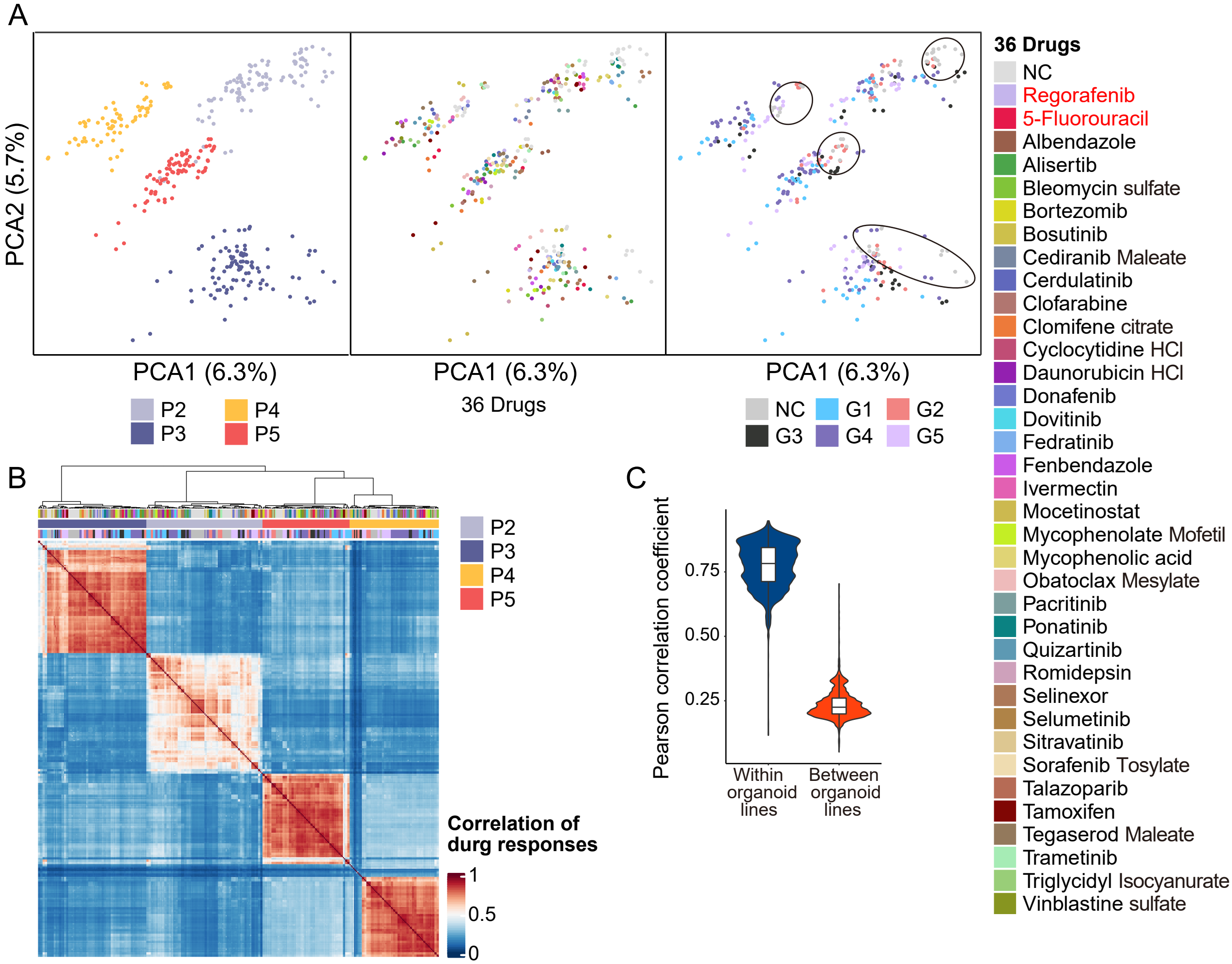

A
PCA2 (5.7%)
PCA1 (6.3%)
P2
P4
P3
P5
36 Drugs
NC
G1
G2
G3
G4
G5
36 Drugs
NC
Regorafenib
5-Fluorouracil
Albendazole
Alisertib
Bleomycin sulfate
Bortezomib
Bosutinib
Cediranib Maleate
Cerdulatinib
Clofarabine
Clomifene citrate
Cyclocytidine HCl
Daunorubicin HCl
Donafenib
Dovitinib
Fedratinib
Fenbendazole
Ivermectin
Mocetinostat
Mycophenolate Mofetil
Mycophenolic acid
Obatoclax Mesylate
Pacritinib
Ponatinib
Quizartinib
Romidepsin
Selinexor
Selumetinib
Sitravatinib
Sorafenib Tosylate
Talazoparib
Tamoxifen
Tegaserod Maleate
Trametinib
Triglycidyl Isocyanurate
Vinblastine sulfate
B
Correlation of durg responses
1
0.5
0
C
Pearson correlation coefficient
0.75
0.50
0.25
Within organoid lines
Between organoid lines

Fig. S5

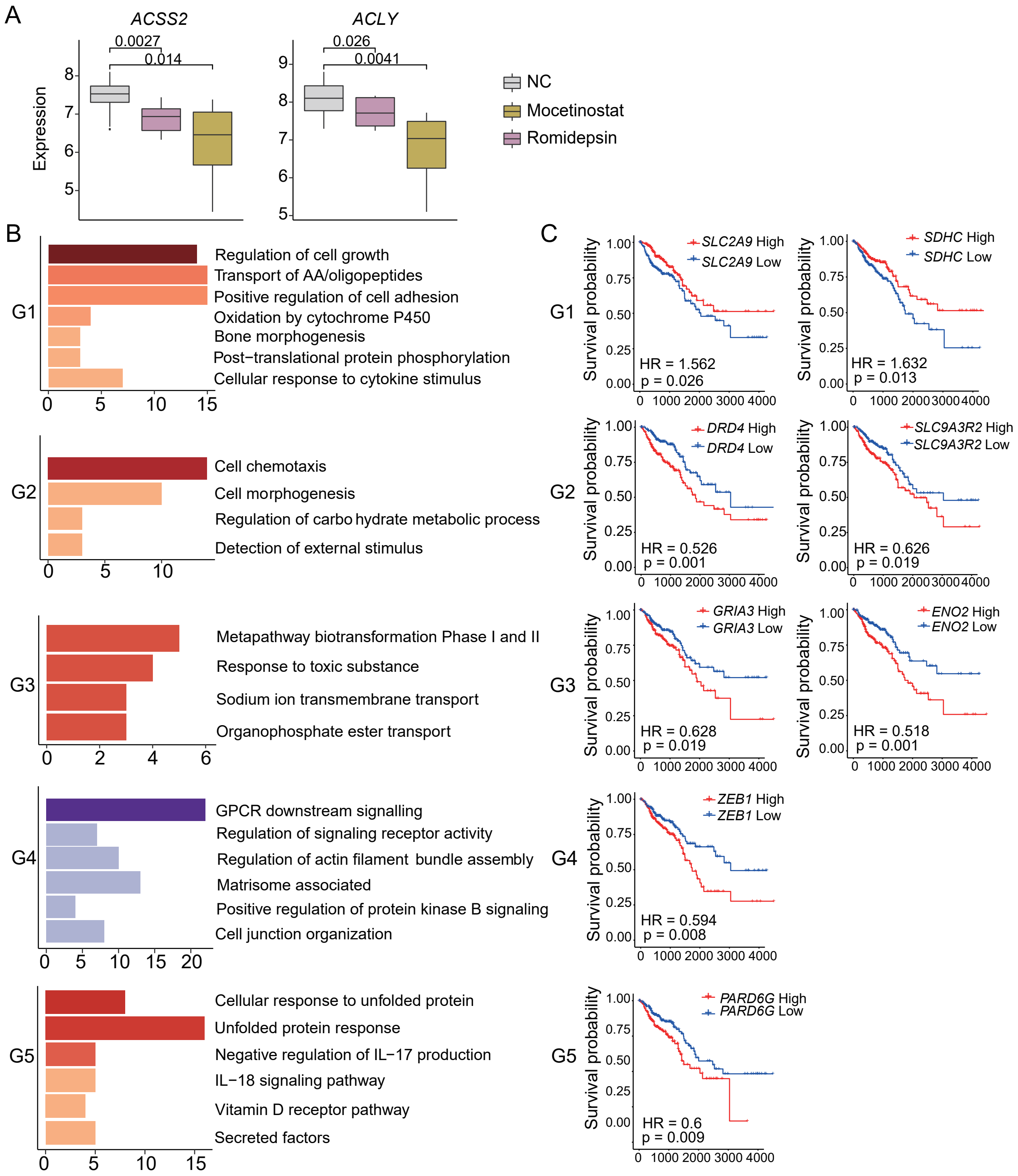

A
ACSS2
ACLY
0.0027
0.014
0.026
0.0041
Expression
NC
Mocetinostat
Romidepsin
B
G1
Regulation of cell growth
Transport of AA/oligopeptides
Positive regulation of cell adhesion
Oxidation by cytochrome P450
Bone morphogenesis
Post−translational protein phosphorylation
Cellular response to cytokine stimulus
G2
Cell chemotaxis
Cell morphogenesis
Regulation of carbo hydrate metabolic process
Detection of external stimulus
G3
Metapathway biotransformation Phase I and II
Response to toxic substance
Sodium ion transmembrane transport
Organophosphate ester transport
G4
GPCR downstream signalling
Regulation of signaling receptor activity
Regulation of actin filament bundle assembly
Matrisome associated
Positive regulation of protein kinase B signaling
Cell junction organization
G5
Cellular response to unfolded protein
Unfolded protein response
Negative regulation of IL−17 production
IL−18 signaling pathway
Vitamin D receptor pathway
Secreted factors
C
Survival probability
SLC2A9 High
SLC2A9 Low
HR = 1.562
p = 0.026
SDHC High
SDHC Low
HR = 1.632
p = 0.013
DRD4 High
DRD4 Low
HR = 0.526
p = 0.001
SLC9A3R2 High
SLC9A3R2 Low
HR = 0.626
p = 0.019
GRIA3 High
GRIA3 Low
HR = 0.628
p = 0.019
ENO2 High
ENO2 Low
HR = 0.518
p = 0.001
ZEB1 High
ZEB1 Low
HR = 0.594
p = 0.008
PARD6G High
PARD6G Low
HR = 0.6
p = 0.009

Fig. S6

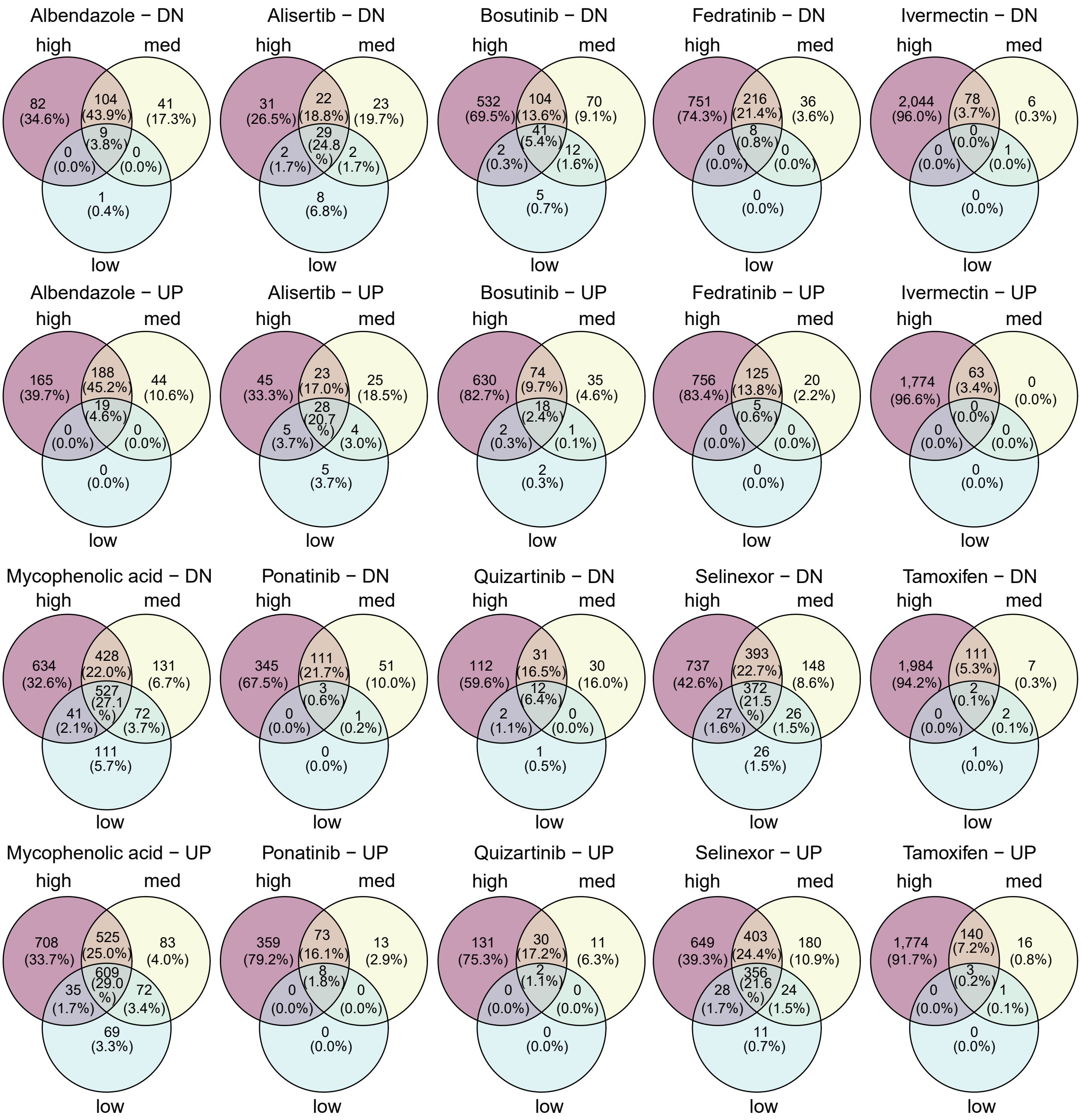
Albendazole − DN
high
med
low
82 (34.6%)
104 (43.9%)
41 (17.3%)
9 (3.8%)
0 (0.0%)
0 (0.0%)
1 (0.4%)
Alisertib − DN
high
med
low
31 (26.5%)
22 (18.8%)
23 (19.7%)
29 (24.8%)
2 (1.7%)
2 (1.7%)
8 (6.8%)
Bosutinib − DN
high
med
low
532 (69.5%)
104 (13.6%)
70 (9.1%)
41 (5.4%)
2 (0.3%)
12 (1.6%)
5 (0.7%)
Fedratinib − DN
high
med
low
751 (74.3%)
216 (21.4%)
36 (3.6%)
8 (0.8%)
0 (0.0%)
0 (0.0%)
0 (0.0%)
Ivermectin − DN
high
med
low
2,044 (96.0%)
78 (3.7%)
6 (0.3%)
0 (0.0%)
0 (0.0%)
1 (0.0%)
0 (0.0%)
Albendazole − UP
high
med
low
165 (39.7%)
188 (45.2%)
44 (10.6%)
19 (4.6%)
0 (0.0%)
0 (0.0%)
0 (0.0%)
Alisertib − UP
high
med
low
45 (33.3%)
23 (17.0%)
25 (18.5%)
28 (20.7%)
5 (3.7%)
4 (3.0%)
5 (3.7%)
Bosutinib − UP
high
med
low
630 (82.7%)
74 (9.7%)
35 (4.6%)
18 (2.4%)
2 (0.3%)
1 (0.1%)
2 (0.3%)
Fedratinib − UP
high
med
low
756 (83.4%)
125 (13.8%)
20 (2.2%)
5 (0.6%)
0 (0.0%)
0 (0.0%)
0 (0.0%)
Ivermectin − UP
high
med
low
1,774 (96.6%)
63 (3.4%)
0 (0.0%)
0 (0.0%)
0 (0.0%)
0 (0.0%)
0 (0.0%)
Mycophenolic acid − DN
high
med
low
634 (32.6%)
428 (22.0%)
131 (6.7%)
527 (27.1%)
41 (2.1%)
72 (3.7%)
111 (5.7%)
Ponatinib − DN
high
med
low
345 (67.5%)
111 (21.7%)
51 (10.0%)
3 (0.6%)
0 (0.0%)
1 (0.2%)
0 (0.0%)
Quizartinib − DN
high
med
low
112 (59.6%)
31 (16.5%)
30 (16.0%)
12 (6.4%)
2 (1.1%)
0 (0.0%)
1 (0.5%)
Selinexor − DN
high
med
low
737 (42.6%)
393 (22.7%)
148 (8.6%)
372 (21.5%)
27 (1.6%)
26 (1.5%)
26 (1.5%)
Tamoxifen − DN
high
med
low
1,984 (94.2%)
111 (5.3%)
7 (0.3%)
2 (0.1%)
0 (0.0%)
2 (0.1%)
1 (0.0%)
Mycophenolic acid − UP
high
med
low
708 (33.7%)
525 (25.0%)
83 (4.0%)
609 (29.0%)
35 (1.7%)
72 (3.4%)
69 (3.3%)
Ponatinib − UP
high
med
low
359 (79.2%)
73 (16.1%)
13 (2.9%)
8 (1.8%)
0 (0.0%)
0 (0.0%)
0 (0.0%)
Quizartinib − UP
high
med
low
131 (75.3%)
30 (17.2%)
11 (6.3%)
2 (1.1%)
0 (0.0%)
0 (0.0%)
0 (0.0%)
Selinexor − UP
high
med
low
649 (39.3%)
403 (24.4%)
180 (10.9%)
356 (21.6%)
28 (1.7%)
24 (1.5%)
11 (0.7%)
Tamoxifen − UP
high
med
low
1,774 (91.7%)
140 (7.2%)
16 (0.8%)
3 (0.2%)
0 (0.0%)
1 (0.1%)
0 (0.0%)

Fig. S7

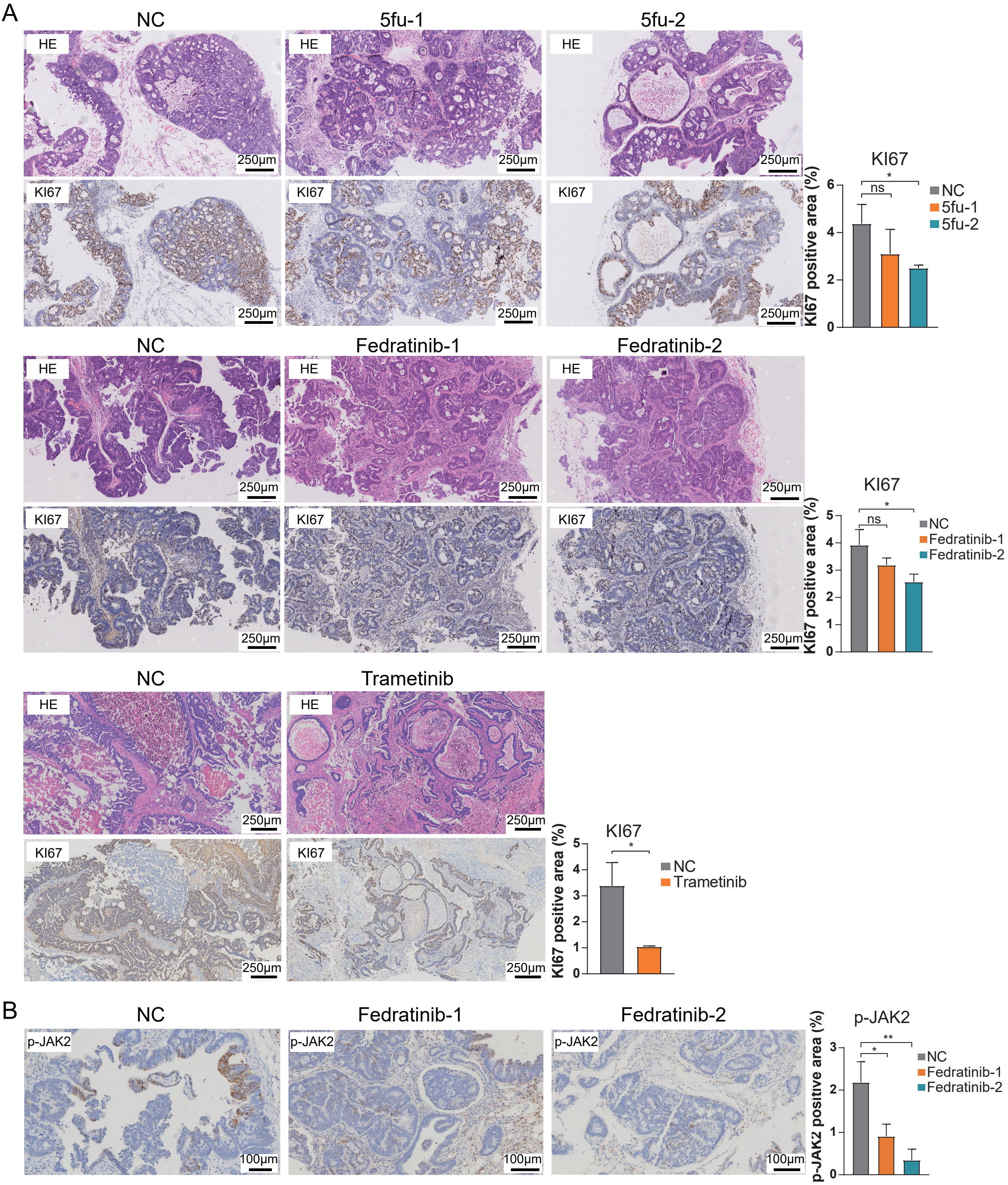
A
NC
5fu-1
5fu-2
HE
KI67
250μm
KI67
KI67 positive area (%)
ns
*
NC
5fu-1
5fu-2
NC
Fedratinib-1
Fedratinib-2
KI67
ns
*
NC
Fedratinib-1
Fedratinib-2
NC
Trametinib
KI67
*
NC
Trametinib
B
NC
Fedratinib-1
Fedratinib-2
p-JAK2
100μm
p-JAK2
p-JAK2 positive area (%)
*
**
NC
Fedratinib-1
Fedratinib-2

Fig. S8

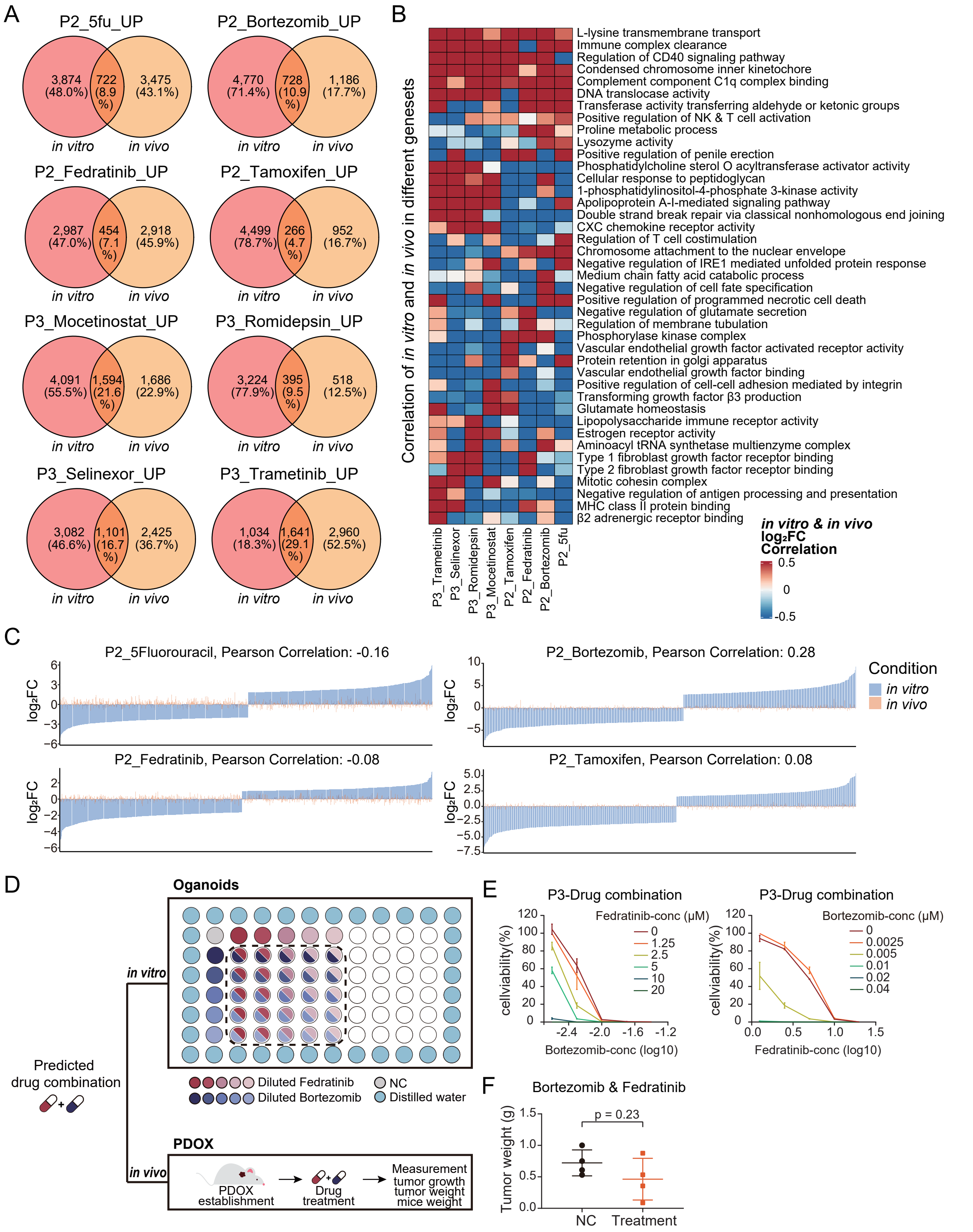
A
P2_5fu_UP
3,874 (48.0%) 722 (8.9 %) 3,475 (43.1%)
P2_Bortezomib_UP
4,770 (71.4%) 728 (10.9 %) 1,186 (17.7%)
P2_Fedratinib_UP
2,987 (47.0%) 454 (7.1 %) 2,918 (45.9%)
P2_Tamoxifen_UP
4,499 (78.7%) 266 (4.7 %) 952 (16.7%)
P3_Mocetinostat_UP
4,091 (55.5%) 1,594 (21.6 %) 1,686 (22.9%)
P3_Romidepsin_UP
3,224 (77.9%) 395 (9.5 %) 518 (12.5%)
P3_Selinexor_UP
3,082 (46.6%) 1,101 (16.7 %) 2,425 (36.7%)
P3_Trametinib_UP
1,034 (18.3%) 1,641 (29.1 %) 2,960 (52.5%)
in vitro
in vivo
B
Correlation of in vitro and in vivo in different genesets
L-lysine transmembrane transport
Immune complex clearance
Regulation of CD40 signaling pathway
Condensed chromosome inner kinetochore
Complement component C1q complex binding
DNA translocase activity
Transferase activity transferring aldehyde or ketonic groups
Positive regulation of NK & T cell activation
Proline metabolic process
Lysozyme activity
Positive regulation of penile erection
Phosphatidylcholine sterol O acyltransferase activator activity
Cellular response to peptidoglycan
1-phosphatidylinositol-4-phosphate 3-kinase activity
Apolipoprotein A-I-mediated signaling pathway
Double strand break repair via classical nonhomologous end joining
CXC chemokine receptor activity
Regulation of T cell costimulation
Chromosome attachment to the nuclear envelope
Negative regulation of IRE1 mediated unfolded protein response
Medium chain fatty acid catabolic process
Negative regulation of cell fate specification
Positive regulation of programmed necrotic cell death
Negative regulation of glutamate secretion
Regulation of membrane tubulation
Phosphorylase kinase complex
Vascular endothelial growth factor activated receptor activity
Protein retention in golgi apparatus
Vascular endothelial growth factor binding
Positive regulation of cell-cell adhesion mediated by integrin
Transforming growth factor β3 production
Glutamate homeostasis
Lipopolysaccharide immune receptor activity
Estrogen receptor activity
Aminoacyl tRNA synthetase multienzyme complex
Type 1 fibroblast growth factor receptor binding
Type 2 fibroblast growth factor receptor binding
Mitotic cohesin complex
Negative regulation of antigen processing and presentation
MHC class II protein binding
β2 adrenergic receptor binding
P3_Trametinib
P3_Selinexor
P3_Romidepsin
P3_Mocetinostat
P2_Tamoxifen
P2_Fedratinib
P2_Bortezomib
P2_5fu
in vitro & in vivo log₂FC Correlation
0.5
0
-0.5
C
P2_5Fluorouracil, Pearson Correlation: -0.16
P2_Bortezomib, Pearson Correlation: 0.28
P2_Fedratinib, Pearson Correlation: -0.08
P2_Tamoxifen, Pearson Correlation: 0.08
log₂FC
Condition
in vitro
in vivo
D
Oganoids
in vitro
in vivo
Predicted drug combination
Diluted Fedratinib
Diluted Bortezomib
NC
Distilled water
PDOX
PDOX establishment
Drug treatment
Measurement tumor growth tumor weight mice weight
E
P3-Drug combination
Fedratinib-conc (μM)
0
1.25
2.5
5
10
20
cellviability(%)
Bortezomib-conc (log10)
P3-Drug combination
Bortezomib-conc (μM)
0
0.0025
0.005
0.01
0.02
0.04
Fedratinib-conc (log10)
F
Bortezomib & Fedratinib
p = 0.23
Tumor weight (g)
NC
Treatment
